# Regional inequalities in Brazil and new approaches to measuring population aging

**DOI:** 10.1590/0102-311XEN217725

**Published:** 2026-06-26

**Authors:** Anderson Gonçalves, Luciana Correia Alves

**Affiliations:** 1 Centro Universitário Padre Anchieta, Jundiaí, Brasil.; 2 Universidade Estadual de Campinas, Campinas, Brasil.

**Keywords:** Population, Aging, Health Inequalities, População, Envelhecimento, Desigualdades de Saúde, Población, Envejecimiento, Inequidades en Salud

## Abstract

This study aims to measure and analyze population aging in Brazil by combining chronological age and characteristics of population subgroups according to geographic region and sex. Using these indicators, the study advances the application of the relative age concept based on a physiological indicator related to the aging process. The data analyzed were drawn from the 2015 *Brazilian Longitudinal Study of Aging*. Variables analyzed included age, sex, region of residence (North, Northeast, Central-West, Southeast, and South), handgrip strength, and body mass index. The results indicate that individuals in the North and Northeast experience faster functional aging according to relative age estimates based on handgrip strength. Among women in these regions, the gap between relative and chronological age reaches up to 9.3 years. These findings highlight regional inequalities in aging and support the use of relative age as a composite indicator for monitoring and planning health policies.

## Resumo

Este artigo buscou medir e analisar o envelhecimento da população brasileira através de uma combinação de idade cronológica e características dos subgrupos populacionais de acordo com a região geográfica de residência e o sexo. Com esses indicadores, este estudo avança a aplicação do conceito de idade relativa com base em um indicador fisiológico relacionado ao processo de envelhecimento. Os dados analisados provêm do *Estudo Longitudinal da Saúde dos Idosos Brasileiros* para 2015. As variáveis de interesse foram idade, sexo, região do Brasil (Norte, Nordeste, Centro-oeste, Sudeste e Sul), força de preensão manual e índice de massa corporal. Os resultados indicam que indivíduos nas regiões Norte e Nordeste apresentam envelhecimento funcional mais rápido de acordo com estimativas relativas de idade baseadas na força de preensão manual. Entre as mulheres nessas regiões, a diferença entre idade relativa e cronológica chega a até 9,3 anos. Os resultados destacam desigualdades regionais no envelhecimento e apoiam o uso da idade relativa como um indicador sintético para monitoramento e planejamento de ações de saúde.

## Resumen

Este artículo buscó medir y analizar el envejecimiento de la población brasileña mediante una combinación de edad cronológica y características de los subgrupos poblacionales según la región geográfica de residencia y el sexo. Con estos indicadores se avanza en la aplicación del concepto de edad relativa a partir de un indicador fisiológico relacionado con el proceso de envejecimiento. Los datos analizados provienen del *Estudio Longitudinal de la Salud de los Ancianos Brasileños* para 2015. Las variables de interés fueron la edad, el sexo, la región de Brasil (Norte, Nordeste, Centro-Oeste, Sudeste y Sur), la fuerza de agarre manual y el índice de masa corporal. Los resultados indican que los individuos de las regiones Norte y Nordeste presentan un envejecimiento funcional más rápido de acuerdo con las estimaciones relativas de edad basadas en la fuerza de agarre. Entre las mujeres de estas regiones, la diferencia entre la edad relativa y la cronológica alcanza hasta los 9,3 años. Los resultados indican desigualdades regionales en el envejecimiento y apoyan el uso de la edad relativa como un indicador sintético para monitorear y planificar acciones de salud.

## Introduction

Chronological age is a traditionally useful indicator in the analysis of aging and is typically expressed in complete solar years, representing the time elapsed between the date of birth and the date of the survey ^1^. It also serves as an objective criterion for establishing rights and duties in modern societies, as well as for assigning social roles based on age-related behavioral expectations. The relationship between age and socially assigned roles goes beyond individual and social rights. An important conceptual approach for understanding these relationships is the life course perspective and its associated social pathways ^2^ ^,^ ^3^.

Studies on population aging in Brazil adopt different demographic approaches based on chronological age, including explanations grounded in components of demographic dynamics ^4^ ^,^ ^5^, analyses that combine aging trends with population projections and their implications for public policies ^6^ ^,^ ^7^, and the measurement of regional inequalities in the aging process ^8^. This study contributes to the latter demographic approach by incorporating relative age as a key indicator in assessing regional inequalities in population aging in Brazil.

In the 21st century, the measurement of population aging should no longer be limited to traditional indicators based solely on chronological age. Efforts by Scherbov & Sanderson ^9^ to reconceptualize age have led to two emerging approaches for the measurement of population aging: the prospective approach and the characteristics approach.

The prospective approach complements chronological age with the survival expectancy of the birth cohort, combining chronological age and life expectancy to calculate prospective age. A distinction is drawn between retrospective (chronological) age, which refers to the time lived, and prospective age, which refers to the expected remaining lifetime. This analytical framework, introduced by Sanderson & Scherbov ^10^ ^,^ ^11^, recognizes that individuals can be understood as having two ages: chronological and prospective age, defined by an estimated survival time.

Under chronological age, individuals of the same age have lived the same number of years since birth. Under prospective age, individuals of the same age are expected to have the same number of remaining lifetime. Recent studies have shown that using prospective age to measure and analyze aging in Brazil confirms that some regions are at a more advanced stage of demographic transition than others ^12^. Moreover, when prospective age is used to estimate the percentage of older adults in the Brazilian population, it projects a slower pace of aging than estimates based on chronological age. This difference in population aging rates according to chronological and prospective measures has also been observed in previous studies on Latin America and other emerging countries ^13^ ^,^ ^14^.

The characteristics approach is another framework proposed by Scherbov & Sanderson for measuring and analyzing population aging ^9^ ^,^ ^15^. This approach is important because different populations hold different characteristics at the same chronological age, and because a given birth cohort consists of subgroups that age at different rates. Therefore, the characteristics approach is useful for measuring and comparing aging across distinct populations or among subgroups within a population.

The characteristics approach combines chronological age with other measurable characteristics of individuals within specific population subgroups. Scherbov & Sanderson ^9^ propose creating an alpha age (α-age), which synthesizes chronological age with an additional measurable characteristic. This concept of age is more clearly expressed by the term relative age ^16^. By analogy, just as prospective age results from combining chronological age with life expectancy, relative age results from combining chronological age with another measurable characteristic.

Therefore, this study aims to measure and analyze population aging in Brazil in 2015 by combining chronological age with subgroup characteristics according to region of residence and sex. Using these indicators, the study extends the application of the relative age concept based on a physiological indicator associated with aging. To this end, the relative ages of population subgroups residing in each of the country’s five regions are calculated and analyzed. This study seeks to address the following research question: What regional inequalities in population aging are revealed by the characteristics approach, and what do these differences indicate about the heterogeneity of aging in Brazil? This study contributes to the application of relative age as an operational concept that measures the rate of aging in a context of regional inequalities, thereby supporting the development of a public policy agenda aimed at recognizing and reducing inequities.

## Materials and methods

Data were obtained from a secondary database: the *Brazilian Longitudinal Study of Aging* (ELSI-Brasil), specifically the 2015 baseline wave. We focused on the baseline because the handgrip strength measures central to this analysis (*mf27*, *mf28*, and *mf29*) had substantially higher completion rates in 2015/2016 than in the subsequent 2019/2021 wave, and because the analysis is not affected by the COVID-19 pandemic.

ELSI-Brasil is a nationally representative longitudinal household survey. The sample represents the community-dwelling Brazilian population aged 50 years and older. ELSI-Brasil aims to investigate the dynamics and determinants of population aging in Brazil, as well as challenges posed to social and health systems by this population. To ensure representativeness across urban and rural areas and across municipalities of different sizes, the sampling design combined stratification of primary sampling units (municipalities), census tracts, and households. The final sample included participants residing in 70 municipalities across the country’s five geographic regions ^17^ ^,^ ^18^ ^,^ ^19^.

Relative ages were calculated using data from the 2015 wave of ELSI-Brasil. A physical performance measurement was included: handgrip strength, widely recognized as an objective marker of functioning and a consistent predictor of morbidity and mortality in older adults, particularly in studies on sarcopenia and frailty ^20^ ^,^ ^21^ ^,^ ^22^ ^,^ ^23^ ^,^ ^24^. To ensure representativeness of the target population, sampling weights were incorporated into the analysis. Moreover, aspects of the complex sampling design of the ELSI study were incorporated into the analyses to prevent clustering effects from compromising the estimates accuracy.

Other variables of interest included chronological age (as a discrete variable), sex, region of residence (North, Northeast, Central-West, Southeast, and South), weight, and height. Weight and height were used to calculate body mass index (BMI), which was treated as a continuous variable.

Eligibility criteria for individuals were: (1) completion of three valid handgrip strength measurements, that is, valid entries for variables *mf27*, *mf28*, and *mf29* in the database; (2) having the body weight measurement available (*mf22* > 0); (3) having the height measurement available (*mf13* > 0); (4) having a BMI between 18.4 and 51kg/m^2^; and (5) handgrip strength greater than 4.9kgf. The final sample consisted of 8,492 individuals aged 50 years and older.

These cutoffs were used as data-quality filters to reduce the influence of implausible anthropometric values and extreme handgrip strength observations on the estimation of the age-handgrip relationship used in the sex-specific linear regressions, following the general outlier-screening approach previously described ^15^. The > 5kgf threshold was used only during model-fitting. After estimating regression lines, observations below 5kgf were reintroduced in the final stage (relative age computation), as such low values may reflect genuine functional limitation rather than measurement error.

For each individual in the sample, the average of the three handgrip strength measurements (variables *mf27*, *mf28*, and *mf29*) was calculated. After stratifying data by sex, median grip strength values ​​were estimated for each region of the country across six age groups: 50-52, 53-57, 58-62, 63-67, 68-72, and 73-77 years. The median value for each age group was taken as representative of its central age. This procedure was adopted to address the limited number of observations by simple age, which could compromise the stability of the estimates, particularly given the simultaneous stratification by sex and region.

We first assessed the quality of age reporting using the Whipple Index and the Myers Index. The results indicated satisfactory age reporting in the sample.

The calculation of relative age requires that the standard population values be available as a continuous variable, expressed in simple ages, and correlated with chronological age. To meet these requirements, the national median handgrip strength (Brazil), estimated by simple chronological age, was used as the standard. Accordingly, two sex-stratified, survey-weighted simple linear regression models were fitted using all eligible individual-level observations. The dependent variable was individual mean handgrip strength (kgf), calculated as the average of the three trials (*mf27*, *mf28*, and *mf29*), and the independent variable was chronological age (years) ^25^.

Due to the presence of discrepant values at older ages, the simple linear regression model for men was adjusted to ages 50-85 years, whereas the model for women was adjusted to ages 50-80 years. Before adjusting the model, we verified that handgrip strength met the assumption of normal distribution.

For model fitting, key assumptions (linearity and homoscedasticity) were evaluated using residual diagnostics and graphical inspection. Model fit was summarized by the coefficient of determination and residual plots. The significance level was set at 5%. Details on these diagnostics are provided in the Supplementary Material (https://cadernos.ensp.fiocruz.br/static//arquivo/suppl-e00217725_3030.pdf).

(1) $$Y_{i}=\beta_{0}+\beta_{1}X_{i}+\varepsilon_{i}$$

in which *Y*_*i*_ denotes grip strength, *X*_*i*_ denotes chronological age, and *ε*_*i*_ represents the model error term, capturing variation in strength not explained by age. These two simple linear regression models, fitted separately by sex (men and women) for Brazil, served as references to calculate relative age. Specifically, the slope of the linear regression for Brazil was used as the standard to estimate the relative ages of age subgroups in each of the five regions. The estimated slope for the standard population (Brazil) was expressed as continuous values across simple age, separately for men and women. The regression models were used solely to construct the national, sex-specific reference lines required for relative age mapping, while regional comparisons were based on observed age-specific medians.

After obtaining grip strength results for Brazil (standard population) based on simple linear regression, observed median grip strength values were used for each subgroup of interest (index age groups within each region). The observed data were organized into tables to calculate the relative age of each population stratum at five chronological ages: 55, 60, 65, 70, and 75 years. This procedure made it possible to determine the relative age corresponding to each population stratum at these specific chronological ages.

To calculate the relative age of an index age group using the standard population, grip strength of the index age group is first identified. Next, the chronological age at which the standard population reaches the same handgrip strength value is determined. For example, among men residing in the Southeast, index group 60 (individuals aged 58-62 years) has a median handgrip strength of 35.7kgf. This value is attributed to men aged 60 years residing in the Southeast. When observing the survey-weighted regression line for men in Brazil (standard population), the predicted handgrip strength was 35.9kgf at 56 and 35.5kgf at 57 years of chronological age. Therefore, the relative age of index group 60 in the Southeast lies between 56 and 57 years. Linear interpolation was used to estimate the corresponding chronological age. Relative age was calculated by linear interpolation, as shown in Equation 2.

(2) $$\text{Relative age}=\text{x₀}+\left[\frac{\left(y-y_{0}\right)}{\left(y_{1}-y_{0}\right)}\right]\left(x_{1}-x_{0}\right)$$

in which *x*_*0*_ is the lower-bound chronological age of the standard population; *x*_*1*_ is the upper-bound chronological age of the standard population; *y* is the handgrip strength of the index age group; *y*_*0*_ is the handgrip strength of the standard population at age *x*_*0*_ ; and *y*_*1*_ is the handgrip strength of the standard population at age *x*_*1*_ .

Based on the values presented, we have:

(3) x₀=56; y₀=35.9

(4) x₁=57; y₁=35.5

(5) y=35.7

Applying these values to the equation, we have:

(6) $$\text{Relative age}=\text{56}+\left[\frac{\left(\text{35.7}-\text{35.9}\right)}{\left(\text{35.5}-\text{35.9}\right)}\right]\left(\text{57}-\text{56}\right)=\text{56.5}$$

Based on this example, it can be observed that men residing in the Southeast at 60 years of chronological age have, on average, a handgrip strength equivalent to that of 56.5-year-old men in the standard population. Thus, the relative age of 60-year-old men in Southeast Brazil is estimated at 56.5 years. This indicates that individuals in this index group exhibit functional characteristics consistent with those of men who are, on average, younger than Brazilians of the same chronological age.

All analyses were performed in R, version 4.5.0 (http://www.r-project.org), using the RStudio interface (https://rstudio.com/). The complex sampling design was specified with the *survey* and *srvyr* packages (strata, primary sampling unit, and calibrated weights), from which estimates of means, medians, standard deviations (SD), and weighted proportions were obtained. Data preparation and organization were performed with *DemoTools*, *dplyr*, and *tidyr*. Handgrip strength was modeled as a function of age estimated by linear regression (*svyglm*) stratified by sex. Relative ages were estimated by linear interpolation (*approx*) of the predicted curves.

ELSI-Brasil was approved by the Research Ethics Committee of the René Rachou Institute, Oswaldo Cruz Foundation, and is registered on Plataforma Brasil (https://plataformabrasil.saude.gov.br; approval certificate n. 34649814.3.0000.5091). All participants provided written informed consent for each research procedure and authorized access to corresponding secondary databases.

## Results

The distribution of the sample by sex, geographic region, age group, and mean BMI is presented in Table 1. All estimates were calculated considering the sampling design of the survey and are therefore representative of the Brazilian population aged 50 years and older in 2015. Women comprised most of the sample (55.8%), and the Southeast was the predominant region of residence (41.5%). The mean age of the sample was 62.1 chronological years (SD ± 9.4).

**Table 1:** Distribution of participants by key sociodemographic characteristics. *Brazilian Longitudinal Study of Aging* (ELSI-Brasil), Brazil (N = 8,492).

Category	n	%	Mean BMI	SD
Sex				
Female	4,739	55.8	27.26	4.54
Male	3,753	44.2	28.80	5.39
Region				
Southeast	3,522	41.5	28.42	5.21
Northeast	2,221	26.2	27.30	4.78
South	1,192	14.0	28.33	4.92
Central-West	854	10.1	27.96	5.22
North	703	8.3	27.88	5.07
Age group (years)				
50-52	1,158	13.6	28.43	5.24
53-57	1,861	21.9	28.46	5.17
58-62	1,567	18.5	28.17	5.08
63-67	1,311	15.4	28.17	5.26
68-72	998	11.8	27.17	4.79
73-77	794	9.3	27.37	4.84
78-82	466	5.5	27.61	4.49
83-87	232	2.7	27.06	4.34
88+	105	1.2	25.00	3.40

BMI: body mass index; SD: standard deviation.

Source: prepared by the authors, according to data from ELSI-Brasil (2015/2016).

The interpretation of BMI differs between adults and older adults because age-related changes in body composition and height influence this indicator. Therefore, BMI was not used as an outcome variable in this study but rather as an operational criterion to define the sample (by excluding implausible values) and to characterize it descriptively. Mean BMI values ​​were very similar across regions, suggesting anthropometric comparability and minimizing confounding due to systematic differences in body size. Thus, the use of handgrip strength as a functional marker to estimate relative age is justified, based on the principle that strength is related to muscle mass and function and declines with aging. The similarity in BMI across regions further supports this choice, as regional differences in strength are unlikely to reflect differences in body mass alone.

**Figure 1:**
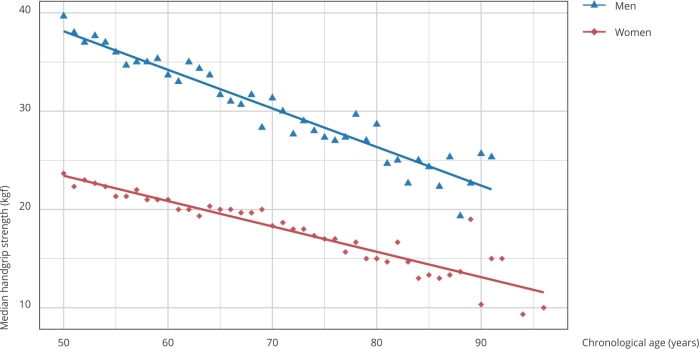
Dispersion of handgrip strength by sex among Brazilians aged 50-100 years in 2015/2016. *Brazilian Longitudinal Study of Aging* (ELSI-Brasil).

Source: prepared by the authors, according to data from ELSI-Brasil (2015/2016).

A graphical analysis of the relationship between chronological age and handgrip strength reveals a nearly linear decline in both sexes, particularly within the 50-85 age range (Figure 1). Within this interval, median strength decreases relatively steadily, whereas beyond age 85 the dispersion of data points increases, indicating greater interindividual variability. The data also reveal differences between men and women. Men experience a steeper decline in strength with advancing age, while women exhibit a more gradual decline.

The parameters obtained from the adjusted linear regression models for both sexes confirm this steeper decline in handgrip strength among men, as shown in Equations 7 and 8:

(7) $$Women:{Ŷ}_{i}=33.1138619-0.2084293xX_{i}+\varepsilon_{i}$$

(8) $$Men:{Ŷ}_{i}=58.0460322-0.3960454xX_{i}+\varepsilon_{i}$$

in which *Ŷ*_*i*_ represents the estimated handgrip strength and *X*_*i*_ corresponds to chronological age. These two simple linear regression models estimated for Brazil were used as the reference to calculate the relative age of the index age subgroups in each region. The adjusted equations for each sex indicate that the angular coefficient, representing the annual rate of decline in handgrip strength, is more accentuated for men than for women. Specifically, the model estimates an average reduction of 0.3960kgf per year of life after 50 chronological years for men and a more gradual decline of 0.2084kgf per year of life for women. This difference in slope reinforces the graphical observation that, although both sexes experience a progressive loss of strength with aging, the rate of decline is greater among men.

Table 2 shows the observed data used to calculate relative ages: medians handgrip strength values, followed by their respective SD according to age group, sex, and region.

**Table 2:** Handgrip strength, median, by age group, sex, and region. *Brazilian Longitudinal Study of Aging* (ELSI-Brasil), Brazil.

Sex/Age group (years)	Central age (years)	Handgrip strength (kgf) [median (SD)]				
		Northeast	North	Central-West	Southeast	South
Men						
53-57	55	32.3 (8.0)	32.7 (7.4)	38.3 (8.8)	38.3 (9.0)	37.0 (9.4)
58-62	60	31.0 (8.8)	31.7 (6.8)	36.7 (9.0)	35.7 (7.4)	35.3 (8.7)
63-67	65	29.7 (7.9)	30.3 (5.3)	34.3 (7.2)	33.0 (7.7)	34.0 (7.5)
68-72	70	28.0 (5.8)	27.7 (5.2)	29.7 (6.3)	31.0 (6.9)	33.7 (6.4)
73-77	75	26.0 (8.6)	27.0 (4.4)	27.0 (6.5)	28.0 (7.1)	31.0 (7.5)
78-82	80	25.7 (6.5)	24.3 (7.3)	29.7 (6.6)	28.0 (6.7)	27.0 (5.9)
Women						
53-57	55	20.3 (5.9)	21.0 (4.8)	21.7 (5.8)	22.0 (6.0)	23.3 (5.3)
58-62	60	19.7 (5.1)	18.7 (5.0)	21.7 (5.4)	21.0 (5.4)	21.0 (5.5)
63-67	65	18.7 (5.1)	18.7 (5.5)	20.7 (4.9)	20.3 (5.3)	21.0 (5.2)
68-72	70	17.7 (5.2)	17.3 (4.8)	20.7 (5.2)	20.0 (5.6)	19.3 (4.8)
73-77	75	15.3 (5.0)	15.3 (3.5)	17.3 (3.8)	17.7 (4.7)	18.0 (5.6)
78-82	80	15.0 (4.2)	14.0 (3.6)	14.3 (4.3)	16.0 (5.2)	16.3 (5.2)

Source: prepared by the authors, according to data from ELSI-Brasil (2015/2016).

The analysis of these data makes it possible to identify regional differences in the functioning of Brazilians aged 50 years and older. The highest strength values for both men and women were found in the South, Southeast, and Central-West, whereas the lowest values were found in the North and Northeast. These regional variations may reflect not only physiological differences but also broader contextual factors, including socioeconomic conditions and lifestyle characteristics. The assessment of regional age-specific medians provides the basis for calculating relative ages, thereby enabling comparisons between regional subgroups and the national standard population.

To capture the heterogeneity in the aging process among Brazilians from different regions but within the same birth cohort, the use of relative age is a pertinent analytical approach. By incorporating functional characteristics, such as handgrip strength, rather than relying solely on chronological age, we identify a measure of variations in aging associated with socioeconomic and regional contexts. Thus, an analysis based on relative age provides a better understanding of inequalities in aging, revealing that individuals of the same chronological age may have different functional stages, depending on the structural conditions and life-course exposures that have shaped their trajectories. Moreover, the use of relative age makes it possible to also analyze whether populations classified as older under traditional or prospective approaches are composed of individuals who exhibit relatively younger or older functional characteristics than the national average for individuals in other regions of the country.

Table 3 shows the estimates of relative age (in years) by sex, region, and chronological age. Each relative-age estimate is accompanied, in parentheses, by the difference between relative and chronological age. Positive values indicate that individuals in a given region have functional characteristics associated with older age compared with the national average for Brazilians of the same chronological age. In other words, their estimated relative age exceeds their chronological age. This pattern is observed for both sexes in the North and Northeast regions.

**Table 3:** Relative age, with difference from chronological age in parentheses, by chronological age, sex, and region. *Brazilian Longitudinal Study of Aging* (ELSI-Brasil), Brazil.

Sex/Chronological age (years)	Relative age (years)				
	Northeast	North	Central-West	Southeast	South
Men					
55	63.7 (+8.7)	64.1 (+9.1)	50.0 (-5.0)	50.0 (-5.0)	53.1 (-1.9)
60	67.3 (+7.3)	66.6 (+6.6)	54.0 (-6.0)	56.5 (-3.5)	57.3 (-2.7)
65	73.6 (+8.6)	70.0 (+5.0)	59.9 (-5.1)	63.2 (-1.8)	60.7 (-4.3)
70	75.6 (+5.6)	76.7 (+6.7)	71.7 (+1.7)	68.3 (-1.7)	61.6 (-8.4)
75	79.1 (+4.1)	78.4 (+3.4)	78.4 (+3.4)	75.9 (+0.9)	68.3 (-6.7)
Women					
55	61.3 (+6.3)	58.1 (+3.1)	54.9 (-0.1)	53.3 (-1.7)	50.0 (-5.0)
60	64.5 (+4.5)	69.3 (+9.3)	54.9 (-5.1)	58.1 (-1.9)	58.1 (-1.9)
65	69.3 (+4.3)	69.3 (+4.3)	59.7 (-5.3)	61.3 (-3.7)	58.1 (-6.9)
70	74.1 (+4.1)	75.7 (+5.7)	59.7 (-10.3)	62.9 (-7.1)	66.1 (-3.9)
75	80.0 (+5.0)	80.0 (+5.0)	75.7 (+0.7)	74.1 (-0.9)	72.5 (-2.5)

Source: prepared by the authors, according to data from ELSI-Brasil (2015/2016).

Based on the data in Table 3, individuals from the North and Northeast regions have handgrip strength characteristics that indicate faster aging compared with the average for Brazilians of the same chronological age. For instance, men aged 60 years in the Northeast present a median handgrip strength corresponding to a relative age of 67.3 years, meaning they are 7.3 years older in functional terms than the average Brazilian population of the same sex and chronological age.

When the estimated relative age for a region is lower than its chronological age, the resulting difference is negative (as shown in parentheses in Table 3), indicating that the region is aging at a slower rate than the average for Brazilians of the same chronological age and sex. For instance, women aged 65 years living in the South have an estimated relative age of 58.1 years, meaning they have a handgrip strength that corresponds to 6.9 years less than the average for Brazilian women of the same chronological age.

Relative ages in the Southeast Region are predominantly lower than chronological ages, suggesting comparatively more favorable functional profiles. In contrast, no consistent pattern is observed in the Central-West, as some cohorts display relatively younger functional characteristics while others have relatively older profiles than the national average for Brazilians of the same chronological age.

A comparison between men and women reveals differences in the pattern of relative ages across cohorts. Among men, the gaps between relative and chronological age are greater at younger ages (55-60) and become less consistent at older ages (65-75). Among women, the differences are heterogeneous across ages, with larger contrasts emerging around 60-70.

## Discussion

In 2018, a review of Brazil’s population projections conducted by the Brazilian Institute of Geography and Statistics (IBGE, acronym in Portuguese) estimated that the country would have 30.2 million older adults (aged 60+ years) in 2020, corresponding to 14.3% of the Brazilian population in that year ^26^ - a projection that was largely confirmed by the initial results of the 2022 population census ^27^. According to the most recent IBGE population projections issued in 2024, the absolute number of people aged 60 years and over is projected to more than double by 2070, reaching an estimated 75.3 million individuals, or roughly 38.8% Brazil’s total population ^28^.

These data describe population aging in Brazil at an aggregate level but are insufficient to fully inform decision-making in both public and private sectors. This limitation stems from the growing heterogeneity among individuals aged 60 years and older, changes in the perception of age over time resulting from the increase in life expectancy, and socioeconomic differences across Brazil’s five regions. In other words, the population aged 60 years and older should be assessed using approaches that account for this heterogeneity, recognizing differences in functioning, needs, characteristics, and lifestyles. Studies aimed at measuring and assessing aging at the population level cannot be separated from aging at the level of individuals and their characteristics.

Brazil’s large territorial size and regional differences in socioeconomic development are determinants of inequalities in the demographic transition process and, consequently, in the stage of aging of each of the five regions. Thus, it is not possible to analyze a single demographic transition at the national level. Rather, the occurrence of multiple transitions and corresponding differences in the stage of aging must be recognized ^7^ ^,^ ^29^.

Based on data from 1950 to 2010 using traditional measures based on chronological age, Vasconcelos & Gomes ^8^ describe the Brazilian demographic transition and conclude that the different regions of the country have distinct rates and levels of mortality and fertility, strengthening the argument that the national transition is neither homogeneous nor simultaneous. The decline in mortality and fertility began in the South, Southeast, and Central-West regions, while the North and Northeast remained at higher levels.

Population projections for 2020-2070 indicate that all regions of Brazil are undergoing a transition in their age structure; that is, population aging is a widespread, although not homogeneous, trend. Using traditional indicators based on chronological age and prospective measures based on life expectancy, Gonçalves & Alves ^12^ conclude that the North, Northeast, and Central-West are at relatively later stages of the aging process. Whether based on the median age of the population, the proportion of older adults, or the old-age dependency ratio, the South and Southeast have relatively older populations than the other regions.

Therefore, prospective and traditional measures provide information on differences in the stage of aging among the five regions of Brazil. This information could suggest that investments in public policies directed at older adults should be concentrated in regions with the oldest populations, namely the South and Southeast. However, information on relative age draws attention to the fact that residents of the North and Northeast experience unfavorable conditions in the aging process. In other words, individuals in these two regions, on average, have characteristics of older individuals than other Brazilians of the same chronological age.

Using the characteristics approach, the contrast in relative ages across regions reveals that individuals in the North and Northeast, on average, have characteristics of relatively older individuals than those in other regions of the same chronological age. For instance, at 65 chronological years, the relative age of women is 69.3 years in the North and Northeast, but 58.1 years in the South, representing a difference of 11.2 years. Similar differences are observed in the analysis of men. These regional differences in relative ages indicate significant inequalities that affect the rate of aging among Brazilians.

Some regions have infrastructure and socioeconomic conditions that favor aging at a slower pace than the national average. This means that the relative age of individuals in these regions will tend to be below their chronological age. Thus, relative age is a metric that can be used to measure regional inequalities in aging and, consequently, to guide public policies aimed at achieving greater equity in this process. Future studies could expand the use of the characteristics approach by including biomarkers other than handgrip strength that are related to the aging process. They could also examine how sociodemographic composition contributes to regional differences in relative age, helping to disentangle compositional from contextual mechanisms.

There is a common assertion linking the phenomenon of Brazilian population aging with the level of wealth that the country is capable of generating. According to this perspective, Brazil is aging before becoming rich. New approaches to measuring aging in 21st-century Brazil provide complementary perspectives to this debate. First, the prospective approach suggests that the pace of population aging may be slower than that projected by traditional measures, since increases in life expectancy alter the perception of age over time ^12^. Second, the characteristics approach highlights regional inequalities that affect the aging process, by measuring differences in relative age and identifying priority regions for public policies aimed at active aging.

Implicitly, the prospective approach is a useful tool for planning social security reforms, as increased life expectancy has been used to justify several reforms in Brazil ^12^. However, this national-level approach does not enable a proper identification of regional inequalities in the aging process. Moreover, studies analyzing healthy life expectancy in Brazil have shown limitations of using life expectancy alone as an indicator of health conditions, as increased life expectancy may be accompanied by a reduction in the average number of years lived without limitations in daily activities ^30^.

Thus, national policies must consider not only chronological and prospective ages, but also relative age across different population subgroups, as a measure of inequalities in the rate of aging in each region of the country.

The combination of these approaches to measuring aging offers a useful set of indicators for a more robust analysis of the phenomenon and underscores the importance of developing targeted public policies to reduce inequalities in the aging process and promote social justice.

## Data Availability

The databases used in the study, including extraction codes, analyses, and results, are available in the repository: https://elsi.cpqrr.fiocruz.br/data-access/.

## Material Suplementar

Material Suplementar
