# Supplementary material for: Regional inequalities in Brazil and new approaches to measuring population aging
Source: Cad Saude Publica. 2026 Jun 26;42:e00217725. doi: 10.1590/0102-311XEN217725 (PMC13313696; doi:10.1590/0102-311XEN217725)
Supplement: Material Suplementar [file 1678-4464-csp-42-EN217725-s.pdf]

SUPPLEMENTARY MATERIAL

1. Regional sociodemographic profile of the analytic sample

Supplementary Table 4 presents a descriptive characterization of the analytic sample by region, including sex, age group, area of residence (urban/rural), years of schooling, and race/color.

Table 4. Sociodemographic characteristics of the study sample by region (N = 8,492)

Category	Southeast n (%)	Northeast n (%)	South n (%)	Central-West n (%)	North n (%)
Sex					
Female	2,019 (42.7)	1,219 (45.1)	681 (42.9)	471 (44.8)	349 (50.4)
Male	1,503 (57.3)	1,002 (54.9)	511 (57.1)	383 (55.2)	354 (49.6)
Age Group					
50-52	480 (13.6)	305 (13.7)	148 (12.4)	117 (13.7)	108 (15.4)
53-57	757 (21.5)	478 (21.5)	271 (22.7)	185 (21.7)	170 (24.2)
58-62	668 (19.0)	395 (17.8)	221 (18.5)	159 (18.6)	124 (17.6)
63-67	547 (15.5)	329 (14.8)	205 (17.2)	137 (16.0)	93 (13.2)
68-72	394 (11.2)	294 (13.2)	147 (12.3)	88 (10.3)	75 (10.7)
73-77	330 (9.4)	209 (9.4)	95 (8.0)	89 (10.4)	71 (10.1)
78-82	208 (5.9)	120 (5.4)	59 (4.9)	46 (5.4)	33 (4.7)
83-87	95 (2.7)	59 (2.7)	37 (3.1)	23 (2.7)	18 (2.6)
88+	43 (1.2)	32 (1.4)	9 (0.8)	10 (1.2)	11 (1.6)
Area of residence					
urban	3,271 (92.9)	1,573 (70.8)	982 (82.4)	811 (95.0)	502 (71.4)
rural	251 (7.1)	648 (29.2)	210 (17.6)	43 (5.0)	201 (28.6)
Years of schooling					
4 years	1,766 (50.1)	1,420 (63.9)	587 (49.2)	465 (54.4)	376 (53.5)
8 years	761 (21.6)	345 (15.5)	279 (23.4)	187 (21.9)	154 (21.9)
≥9 years	983 (27.9)	435 (19.6)	320 (26.8)	200 (23.4)	164 (23.3)
Supletivo[1]	4 (0.1)	3 (0.1)	3 (0.3)	0 (0)	0 (0)
Unknown	8 (0.2)	18 (0.8)	3 (0.3)	2 (0.2)	9 (1.3)
Race/Color					
White	1,536 (43.6)	473 (21.3)	861 (72.2)	281 (32.9)	105 (14.9)
Brown	1,348 (38.3)	1,358 (61.1)	251 (21.1)	450 (52.7)	459 (65.3)
Asian	34 (1.0)	24 (1.1)	7 (0.6)	9 (1.1)	7 (1.0)
Black	340 (9.7)	278 (12.5)	41 (3.4)	84 (9.8)	47 (6.7)
Indigenous	70 (2.0)	29 (1.3)	6 (0.5)	15 (1.8)	82 (11.7)
Unknown	194 (5.5)	59 (2.7)	26 (2.2)	15 (1.8)	3 (0.4)

[1]Supletivo refers to Brazil's accelerated adult education modality for completing primary/secondary schooling.
Source: ELSI-Brazil (2015/2016). Prepared by the authors.

This table is provided in the Supplementary Material to address the reviewer's request for regional sociodemographic contextualization, while preserving the focus of the main manuscript on introducing and applying the characteristics approach to derive relative age and describe regional

inequalities in functional aging. Because these sociodemographic distributions are not directly analyzed as determinants within the present study design, we do not discuss them in detail in the main text; instead, we make them available here to support transparency and to motivate future analyses that may explicitly examine compositional versus contextual mechanisms.

2. Survey-weighted linear regression models used to derive the standard handgrip-strength

This section documents the survey-weighted linear models fitted to ELSI-Brazil (Wave 1; 2015/2016) to obtain sex-specific, age-based predicted mean handgrip strength values for the Brazilian standard population. These predicted values were used exclusively as a normative reference for the interpolation-based calculation of relative ages (inverse mapping from grip strength to chronological age) in the subsequent regional analyses; the models were not intended for prediction at the individual level.

Data and analytical sample: The analytical dataset included individuals aged 50+ who satisfied the pre-specified eligibility criteria for performance measures (valid height and weight measurements, completion of the three handgrip trials, and no reported arm surgery), followed by BMI-based exclusions (BMI < 18.5 and BMI > 50). Handgrip strength was defined as the within-person mean of the three trials (mf27, mf28, mf29). For regression modeling, ages were restricted to 50–85 for men and 50–80 for women to align with the study's analytic scope and to avoid sparsity at the oldest ages.

Survey design and estimation approach: All estimates accounted for ELSI's complex sampling design. A survey design object was defined with primary sampling units (upa), strata (estrato), and the calibrated sampling weight (peso_calibrado). Sex-stratified designs were then obtained by subsetting the survey design to each sex and the corresponding age range. Linear models were estimated using svyglm with a Gaussian family, yielding design-based standard errors and inference.

Model results (output R)

```
fit_m <- svyglm(GRIP_STRENGTH ~ idade, design = des_m, family = gaussian())
> fit_f <- svyglm(GRIP_STRENGTH ~ idade, design = des_f, family = gaussian())
>
> cat("\n--- svyglm (Men, 50–85) ---\n")

--- svyglm (Men, 50–85) ---
> print(summary(fit_m)$coefficients)
             Estimate Std. Error   t value      Pr(>|t|)
(Intercept) 58.0460322 1.71440800  33.85777 4.366236e-100
idade       -0.3960454 0.02502189 -15.82796  1.471573e-40
> print(confint(fit_m))
                2.5 %     97.5 %
(Intercept) 54.6710008 61.4210635
idade       -0.4453042 -0.3467866
>
> cat("\n--- svyglm (Women, 50–80) ---\n")

--- svyglm (Women, 50–80) ---
> print(summary(fit_f)$coefficients)
             Estimate Std. Error   t value     Pr(>|t|)
(Intercept) 33.1138619 1.15316228  28.71570 4.787117e-83
idade       -0.2084293 0.01919938 -10.85605 6.268268e-23
> print(confint(fit_f))
                2.5 %     97.5 %
(Intercept) 30.8432565 35.3844672
idade       -0.2462334 -0.1706253
> # ------------------------------------------------------------
> # Weighted R-squared (descriptive) using fitted values and weights
> # Note: not a formal "design-based R^2", but a useful weighted GOF summary.
> # ------------------------------------------------------------
>
> weighted_r2 <- function(fit){
+   y <- model.response(model.frame(fit))
+   w <- weights(fit)
+   yhat <- fitted(fit)
+
+   ybar <- weighted.mean(y, w, na.rm = TRUE)
+   sst  <- sum(w * (y - ybar)^2, na.rm = TRUE)
+   sse  <- sum(w * (y - yhat)^2, na.rm = TRUE)
+
+   1 - (sse / sst)
+ }
>
> r2_m <- weighted_r2(fit_m)
> r2_f <- weighted_r2(fit_f)
>
> cat("\nWeighted R^2 (descriptive):\n")

Weighted R^2 (descriptive):
> cat("Men   (50–85):", round(r2_m, 3), "\n")
Men   (50–85): 0.149
> cat("Women (50–80):", round(r2_f, 3), "\n")
Women (50–80): 0.084
```

In both sexes, the age slope is negative and statistically different from zero under the sampling design ($p < 0.001$), indicating a design-based association between age and mean handgrip strength. However, the strength of the linear association is modest.

Association strength and descriptive goodness-of-fit: To summarize the association strength, we computed the design-based Pearson correlation coefficient between age and handgrip strength using svyvar. In addition, we report a descriptive, weight-based R^2 computed from fitted values and survey weights. This weighted R^2 is not a formal design-based R^2, but it is useful to convey the limited variance explained by age alone. Importantly, these summaries are reported only to characterize the strength of the age–handgrip gradient and the dispersion around the fitted line; they are not intended to support an explanatory or predictive interpretation. In this study, the linear regression is used strictly as a standardization device to obtain a sex-specific reference mean function, which is then inverted via interpolation to map observed subgroup handgrip levels into relative ages.

Across the survey-weighted models, the linear association between chronological age and mean handgrip strength was modest for both sexes. The design-based Pearson correlation was $r = -0.3856$ among men aged 50–85 and $r = -0.3265$ among women aged 50–80, indicating a moderate negative age gradient. Consistently, the descriptive weighted coefficients of determination were low ($R^2 = 0.149$ for men; $R^2 = 0.084$ for women), showing that age alone explains only a small fraction of the variability in individual handgrip strength. Therefore, these models are not intended for individual-level prediction; rather, they provide a smooth, sex-specific normative reference used to standardize grip-strength values and support the interpolation-based estimation of relative ages.

These results indicate that chronological age alone explains a small fraction of the variation in individual handgrip strength. Accordingly, the linear models should not be interpreted as predictive models. In this study, they serve a narrow and methodological purpose: to generate a smooth, monotonic, sex-specific normative trajectory for the Brazilian standard population, which is required for the interpolation-based computation of relative ages from regional grip-strength summaries.

Residual diagnostics (exploratory): Residual behavior was examined graphically using residuals-versus-fitted plots and normal Q–Q plots computed from the *svyglm* fitted values and residuals. However, because *svyglm* is primarily a tool for design-based estimation and inference, residual diagnostics are interpreted strictly descriptively, and they should not be understood as the same "assumption checking" framework used for ordinary least squares models. To complement this descriptive inspection, we also fitted the analogous unweighted OLS model (ignoring the complex

design) on the same eligible sample and conducted the standard residual diagnostics; these checks indicated that the mean linear trend is adequately captured as a first-order approximation. Overall, mild tail departures (common in large samples) do not affect the intended use of the fitted mean function, which in this study serves only for standardization and interpolation in the relative-age mapping procedure.

Reference:

1. Frederiksen, H., Hjelmborg, J., Mortensen, J., McGue, M., Vaupel, J. W., & Christensen, K. (2006). Age trajectories of grip strength: Cross-sectional and longitudinal data among 8,342 Danes aged 46 to 102. *Annals of Epidemiology, 16*(7), 554–562.

R code (Supplementary Material)

```
0001  # Context and rationale for this script
0002
0003  # This analysis focuses on handgrip strength in ELSI-Brazil. During
exploratory
0004  # checks, Wave 1 (2015/2016) appeared more internally consistent than Wave
2
0005  # (2019/2021) specifically for handgrip measures (mf27–mf29). We did not
find
0006  # official documentation describing fieldwork challenges, but the publicly
0007  # referenced fieldwork window for Wave 2 (2019–2021) is notably longer
than for
0008  # Wave 1 (2015–2016), which may reflect extended logistics and/or
disruptions.
0009  # We cannot assert causes, but the longer window overlaps the COVID-19
period,
0010  # which could plausibly have affected data collection; this remains a
hypothesis,
0011  # not a confirmed explanation.
0012  #
0013  # Empirically, Wave 1 shows higher completion rates for all three handgrip
trials:
0014  # 95.2% among men and 92.8% among women. For Wave 2, completion rates are
lower
0015  # (see outputs computed below, 81% among men and 81,8% among women). Given
this
0016  # contrast, the workflow is:
0017  #   (1) start with Wave 2 (recency), quantify completion/eligibility, and
0018  #       conduct primary estimates using the declared complex survey
design; then
0019  #   (2) replicate the same pipeline on Wave 1 to assess robustness and
discuss
0020  #        consistency of findings under a dataset with higher trial
completion.
0021
0022  # Note: All estimates account for ELSI's complex sample design
(PSU/strata/weights).
0023  # Where missingness materially affects Wave 2 handgrip measures,
sensitivity
0024  # analyses are reported (e.g., restricting to those with complete trials
versus
0025  # using alternative eligibility rules) to gauge potential selection bias.
0026
0027  ##############################################################################
0028  ##### Working with data from Wave 2 - 2019/2021 ##########################
0029  ##############################################################################
0030
0031  # Loading useful libraries (Step 1)
0032
0033  library(survey)
0034  library(data.table)
0035  library(dplyr)
0036  library(readr)
0037  library(ggplot2)
0038  library(tidyr)
0039  library(srvyr)
0040  library(gridExtra)
0041  library(haven)
0042  library(readxl)
0043  library(writexl)
```

```
0044  library(openxlsx)
0045  library(demotools)
0046
0047  # The ELSI dataset is available on the study website in both CSV and Stata
13 (.dta) formats.
0048  # We chose the .dta version because it preserves variable types and value
labels,
0049  # avoids locale-related parsing issues (decimal comma vs. point,
delimiters, encoding),
0050  # and tends to load more reliably than CSV for large, labelled survey
data.
0051
0052  # Loading the dataset (Step 2)
0053  ELSI <- as.data.frame(read_dta("ELSI_Wave2_2019.dta"))
0054  # We started with the data from the second wave.
0055
0056  # Creating the survey design object “Data”
0057  # This object declares ELSI’s complex sampling design (PSU/UPA, strata,
and final weight),
0058  # so that all estimates computed from it (means, proportions, regressions,
etc.)
0059  # are weighted and therefore representative of the Brazilian population
aged 50 or older.
0060
0061  Data <- ELSI %>%
0062    as_survey_design(ids = upa, strata = estrato, weights = peso_calibrado,
nest = TRUE)
0063
0064
0065  # This block creates “Variables”, a tidy set of derived, factor-labeled
covariates for analysis.
0066  # It maps sex codes (0/1) to labeled categories, defines two age banding
schemes (Idade, Idade2),
0067  # groups schooling and race/color, recodes region and urban/rural without
overwriting the originals,
0068  # and builds performance/eligibility flags from mf* items using explicit,
non-overlapping thresholds.
0069  # A default branch (TRUE ~ NA_character_) sends unexpected codes to NA,
keeping types stable and
0070  # categories coherent. The result is a clean, analysis-ready set of
variables aligned with the
0071  # declared complex survey design, so downstream estimates are
interpretable and consistent.
0072
0073  Variables <- Data %>% mutate(one=1,
0074                               Sexo = factor(ifelse(sexo ==
1,"masculino","feminino"),
0075                                             levels =
c("masculino","feminino",
0076                                                               TRUE ~
NA_character_)),
0077                               Idade = factor(case_when(
0078                                 idade %in% 50:54 ~ "50-54",
0079                                 idade %in% 55:59 ~ "55-59",
0080                                 idade %in% 60:64 ~ "60-64",
0081                                 idade %in% 65:69 ~ "65-69",
0082                                 idade %in% 70:74 ~ "70-74",
0083                                 idade %in% 75:79 ~ "75-79",
0084                                 idade %in% 80:84 ~ "80-84",
0085                                 idade %in% 85:89 ~ "85-89",
0086                                 idade > 89 ~ "90+"),
0087                                             levels = c("50-54","55-59","60-
```

```
64","65-69","70-74","75-79",
                                          "80-84","85-89","90+",
                                          TRUE ~ NA_character_)),
                                Idade2 = factor(case_when(
                                   idade %in% 50:52 ~ "50-52",
                                   idade %in% 53:57 ~ "53-57",
                                   idade %in% 58:62 ~ "58-62",
                                   idade %in% 63:67 ~ "63-67",
                                   idade %in% 68:72 ~ "68-72",
                                   idade %in% 73:77 ~ "73-77",
                                   idade %in% 78:82 ~ "78-82",
                                   idade %in% 83:87 ~ "83-87",
                                   idade > 87 ~ "88+"),
                                              levels = c("50-52","53-57","58-
62","63-67","68-72","73-77",

"78-82","83-87","88+",
                                                TRUE ~ NA_character_)),
                                Escolaridade = factor(case_when(
                                   e22 %in% 1:5 ~ "quatro",
                                   e22 %in% 6:9 ~ "oito",
                                   e22 %in% 10:12 | e22 %in% 14:18 ~ "nove+",
                                   e22 == 13 ~ "supletivo/madureza",
                                   e22 == 99 ~ "não_sabe",
                                   TRUE ~ NA_character_)),
                                Raca_cor = factor(case_when(
                                   e9 == 1 ~ "branca",
                                   e9 == 2 ~ "preta",
                                   e9 == 3 ~ "parda",
                                   e9 == 4 ~ "amarela",
                                   e9 == 5 ~ "indigena",
                                   e9 == 9 ~ "n sabe ou n respondeu",
                                   TRUE ~ NA_character_)),
                                Regiao = factor(case_when(
                                   regiao == 1 ~ "Norte",
                                   regiao == 2 ~ "Nordeste",
                                   regiao == 3 ~ "Sudeste",
                                   regiao == 4 ~ "Sul",
                                   regiao == 5 ~ "Centro-Oeste"),
                                     levels =
c("Sul","Sudeste","Norte","Nordeste","Centro-Oeste",
                                                  TRUE ~ NA_character_)),
                                Zona = factor(case_when(
                                    zona == 1 ~ "urbana",
                                    zona == 2 ~ "rural",
                                    TRUE ~ NA_character_)),

                                medida_1 = factor(case_when(
                                   mf27 == 555 ~ "tentou,mas /n conseguiu",
                                   mf27 == 666 ~ "n tentou",
                                   mf27 == 777 ~ "incapacitado",
                                   mf27 == 888 | mf27 == 8888 ~ "recusou-se",
                                   mf27 < 100 ~ "realizou",
                                   TRUE ~ NA_character_)),
                                 medida_2 = factor(case_when(
                                   mf28 == 555 ~ "tentou,mas /n conseguiu",
                                   mf28 == 666 ~ "n tentou",
                                   mf28 == 777 ~ "incapacitado",
                                   mf28 == 888 | mf28 == 8888 ~ "recusou-se",
                                   mf28 < 100 ~ "realizou",
                                   TRUE ~ NA_character_)),
                                 medida_3 = factor(case_when(
```

```
                                  mf29 == 555 ~ "tentou,mas /n conseguiu",
                                  mf29 == 666 ~ "n tentou",
                                  mf29 == 777 ~ "incapacitado",
                                  mf29 == 888 | mf29 == 8888 ~ "recusou-se",
                                  mf29 < 100 ~ "realizou",
                                  TRUE ~ NA_character_)),
                                medida_AL1 = factor(case_when(
                                  mf13 > 300 ~ "nao_fez",
                                  mf13 < 300 ~ "realizou",
                                  TRUE ~ NA_character_)),
                                medida_PS1 = factor(case_when(
                                  mf22 > 200 ~ "nao_fez",
                                  mf22 < 200 ~ "realizou",
                                  TRUE ~ NA_character_)),
                                elegivel_altura = factor(case_when(
                                  mf13 < 300 ~ "sim",
                                  mf13 > 300 ~ "não",
                                  TRUE ~ NA_character_)),
                                elegivel_peso = factor(case_when(
                                  mf22 < 200 ~ "sim",
                                  mf22 > 200 ~ "não",
                                  TRUE ~ NA_character_)),
                                elegivel_forca = factor(case_when(
                                  mf27 %in% 5:70 ~ "sim",
                                  mf27 > 70 | mf27 < 5 ~ "não",
                                  TRUE ~ NA_character_)))


# create a new object adding mf_X (max valid grip strength across mf27,
mf28, mf29; values > 100 treated as missing)

Variables_mfX <- Variables %>%
  mutate(
    mf_X = pmax(
      ifelse(mf27 <= 100, mf27, NA_real_),
      ifelse(mf28 <= 100, mf28, NA_real_),
      ifelse(mf29 <= 100, mf29, NA_real_),
      na.rm = TRUE
    ),
    mf_X = ifelse(is.infinite(mf_X), NA_real_, mf_X)  # all three > 100 ->
NA
  )


# convert to data.frame (removes the survey/weights context) and check the
number of observations
Variables_mfX_df <- Variables_mfX %>% as.data.frame()

# number of observations (rows)
nrow(Variables_mfX_df)
summary(Variables_mfX_df$mf_X)
Variables_mfX_df$mf_X

# Exploratory stage: defining eligible observations for the study.
# An individual is considered eligible if they meet ALL of the following:
# 1) Has NOT undergone arm surgery
# 2) Completed all three grip-strength measures
# 3) Has a recorded body weight measurement
# 4) Has a recorded body height measurement

# Alternative eligibility - not performed (Wave 2 data constraint):
```

```
0204  # 2a) Has completed at least the first grip-strength measure in Wave 2,
due to missing data
0205
0206
0207
0208
0209  ################################################
0210  # grip-strength measures #####################
0211  ################################################
0212
0213  # Weighted cross-tabs and column percentages by sex (exclude NAs from
denominators)
0214
0215  # ---- First measure (mf27)
0216  d1 <- subset(Variables, !is.na(Sexo) & !is.na(medida_1))  # exclude NAs to
avoid distorted denominators
0217  tab2 <- svytable(~medida_1 + Sexo, design = d1)
0218  tab2
0219  prop.table(tab2, margin = 2) * 100  # column percentages (by sex)
0220
0221  # ---- Second measure (mf28)
0222  d2 <- subset(Variables, !is.na(Sexo) & !is.na(medida_2))
0223  tab3 <- svytable(~medida_2 + Sexo, design = d2)
0224  tab3
0225  prop.table(tab3, margin = 2) * 100  # column percentages (by sex)
0226
0227  # ---- Third measure (mf29)
0228  d3 <- subset(Variables, !is.na(Sexo) & !is.na(medida_3))
0229  tab4 <- svytable(~medida_3 + Sexo, design = d3)
0230  tab4
0231  prop.table(tab4, margin = 2) * 100  # column percentages (by sex)
0232
0233
0234  rm(tab2,tab3,tab4)
0235
0236  # Unweighted counts using the raw data inside the survey design
0237  df <- Variables$variables
0238
0239  # ---- Cross-tab: medida_1 x Sexo (keep all factor levels; show zeros)
0240  tab_m1_unw <- df %>%
0241    count(medida_1, Sexo, .drop = FALSE) %>%         # keep empty
combinations
0242    complete(medida_1, Sexo, fill = list(n = 0))     # fill explicit zeros
0243
0244  # Present as a wide matrix: rows = medida_1, columns = Sexo
0245  m1_wide <- tab_m1_unw %>%
0246    tidyr::pivot_wider(names_from = Sexo, values_from = n)
0247
0248  m1_wide
0249
0250  # ---- medida_2
0251  tab_m2_unw <- df %>%
0252    count(medida_2, Sexo, .drop = FALSE) %>%
0253    complete(medida_2, Sexo, fill = list(n = 0))
0254  m2_wide <- tab_m2_unw %>%
0255    tidyr::pivot_wider(names_from = Sexo, values_from = n)
0256  m2_wide
0257
0258
0259  # ---- medida_3
0260  tab_m3_unw <- df %>%
0261    count(medida_3, Sexo, .drop = FALSE) %>%
```

```
  complete(medida_3, Sexo, fill = list(n = 0))
m3_wide <- tab_m3_unw %>%
  tidyr::pivot_wider(names_from = Sexo, values_from = n)
m3_wide

m1_wide
m2_wide
m3_wide

class(Variables$variables$mf29)
# The initial measurements (mf27 and mf28) were collected more rigorously
than the subsequent ones (mf29).

# Column percentages (unweighted), by sex
m1_pct_unw <- tab_m1_unw %>%
  group_by(Sexo) %>%
  mutate(pct = 100 * n / sum(n)) %>%
  ungroup()

m1_pct_unw




################################################
# variables related to weight and height
################################################

# Height
tab44 <- svytable(~medida_AL1+Sexo, design=Variables)
tab44
proportions(tab44, margin = 2)*100

rm(tab44)
# Weight
tab45 <- svytable(~medida_PS1+Sexo, design=Variables)
tab45
proportions(tab45, margin = 2)*100

rm(tab44,tab45)


################################################
# Selected #####################################
################################################

#Height
tab46 <- svytable(~elegivel_altura+Sexo, design=Variables)
tab46
proportions(tab46, margin = 2)*100

# Weight
tab47 <- svytable(~elegivel_peso+Sexo, design=Variables)
tab47
proportions(tab47, margin = 2)*100

#strenght
tab48<- svytable(~elegivel_forca+Sexo, design=Variables)
tab48
proportions(tab48, margin = 2)*100

rm(tab46,tab47,tab48)
```

```
0323
0324
0325
0326  ######################################################################
0327  ########### Switch to ELSI Wave 1 (2015/2016) ####################
0328  ######################################################################
0329
0330  # From this point onward, we discontinue the use of ELSI Wave 2
(2019/2021) data,
0331  # because the eligibility filters would exclude a substantial share of the
sample,
0332  # undermining the stability and interpretability of the results.
0333
0334  # Clean up: remove all previously created objects before starting the Wave
1 analysis
0335  rm(list = ls())
0336  gc()
0337  closeAllConnections()
0338
0339
0340
0341
0342  ##############################################################################
0343  ##### Working with data from Wave 1 - 2015/2016 ########################
0344  ##############################################################################
0345
0346  # Loading useful libraries (Step 1)
0347
0348  library(survey)
0349  library(data.table)
0350  library(dplyr)
0351  library(readr)
0352  library(ggplot2)
0353  library(tidyr)
0354  library(srvyr)
0355  library(gridExtra)
0356  library(haven)
0357  library(readxl)
0358  library(writexl)
0359  library(openxlsx)
0360
0361  # The ELSI dataset is available on the study website in both CSV and Stata
13 (.dta) formats.
0362  # We chose the .dta version because it preserves variable types and value
labels,
0363  # avoids locale-related parsing issues (decimal comma vs. point,
delimiters, encoding),
0364  # and tends to load more reliably than CSV for large, labelled survey
data.
0365
0366  # Loading the dataset (Step 2)
0367  ELSI_15 <- as.data.frame(read_dta("ELSI_Wave1_2015.dta"))
0368  # We continued with the data from the Wave 1.
0369
0370
0371  ##############################################
0372  # Creating the survey design object “Data”
0373  ##############################################
0374
0375  # This object declares ELSI’s complex sampling design (PSU/UPA, strata,
and final weight),
0376  # so that all estimates computed from it (means, proportions, regressions,
```

```
etc.)
0377  # are weighted and therefore representative of the Brazilian population
aged 50 or older.
0378
0379  Data_15 <- ELSI_15 %>%
0380    as_survey_design(ids = upa, strata = estrato, weights = peso_calibrado,
nest = TRUE)
0381
0382  ##############################################
0383  # This block creates “Variables”, a tidy set of derived, factor-labeled
covariates for analysis.
0384  # It maps sex codes (0/1) to labeled categories, defines two age banding
schemes (Idade, Idade2),
0385  # groups schooling and race/color, recodes region and urban/rural without
overwriting the originals,
0386  # and builds performance/eligibility flags from mf* items using explicit,
non-overlapping thresholds.
0387  # A default branch (TRUE ~ NA_character_) sends unexpected codes to NA,
keeping types stable and
0388  # categories coherent. The result is a clean, analysis-ready set of
variables aligned with the
0389  # declared complex survey design, so downstream estimates are
interpretable and consistent.
0390
0391  # Crating variables and its categories
0392
0393  Variables_2015 <- Data_15 %>% mutate(one=1,
0394                                Sexo = factor(ifelse(sexo ==
1,"masculino","feminino"),
0395                                              levels =
c("masculino","feminino")),
0396                                Idade = factor(case_when(
0397                                  idade %in% 50:54 ~ "50-54",
0398                                  idade %in% 55:59 ~ "55-59",
0399                                  idade %in% 60:64 ~ "60-64",
0400                                  idade %in% 65:69 ~ "65-69",
0401                                  idade %in% 70:74 ~ "70-74",
0402                                  idade %in% 75:79 ~ "75-79",
0403                                  idade %in% 80:84 ~ "80-84",
0404                                  idade %in% 85:89 ~ "85-89",
0405                                  idade > 89 ~ "90+"),
0406                                  levels = c("50-54","55-59","60-64","65-
69","70-74","75-79",
0407                                             "80-84","85-89","90+",
0408                                             TRUE ~ NA_character_)),
0409                                Idade2 = factor(case_when(
0410                                  idade %in% 50:52 ~ "50-52",
0411                                  idade %in% 53:57 ~ "53-57",
0412                                  idade %in% 58:62 ~ "58-62",
0413                                  idade %in% 63:67 ~ "63-67",
0414                                  idade %in% 68:72 ~ "68-72",
0415                                  idade %in% 73:77 ~ "73-77",
0416                                  idade %in% 78:82 ~ "78-82",
0417                                  idade %in% 83:87 ~ "83-87",
0418                                  idade > 87 ~ "88+"),
0419                                  levels = c("50-52","53-57","58-62","63-
67","68-72","73-77",
0420                                             "78-82","83-87","88+",
0421                                             TRUE ~ NA_character_)),
0422                                Escolaridade = factor(case_when(
0423                                  e22 %in% 1:5 ~ "quatro",
0424                                  e22 %in% 6:9 ~ "oito",
```

```
                                  e22 %in% 10:12 | e22 %in% 14:18 ~ "nove+",
                                  e22 == 13 ~ "supletivo/madureza",
                                  e22 == 99 ~ "não_sabe",
                                  TRUE ~ NA_character_)),
                                Raca_cor = factor(case_when(
                                  e9 == 1 ~ "branca",
                                  e9 == 2 ~ "preta",
                                  e9 == 3 ~ "parda",
                                  e9 == 4 ~ "amarela",
                                  e9 == 5 ~ "indigena",
                                  e9 == 9 ~ "n sabe ou n respondeu",
                                  TRUE ~ NA_character_)),
                                Regiao = factor(case_when(
                                  regiao == 1 ~ "Norte",
                                  regiao == 2 ~ "Nordeste",
                                  regiao == 3 ~ "Sudeste",
                                  regiao == 4 ~ "Sul",
                                  regiao == 5 ~ "Centro-Oeste"),
                                  levels =
c("Sul","Sudeste","Norte","Nordeste","Centro-Oeste",
                                                    TRUE ~ NA_character_)),
                                Zona = factor(case_when(
                                  zona == 1 ~ "urbana",
                                  zona == 2 ~ "rural",
                                  TRUE ~ NA_character_)),
                                Renda = factor(case_when(
                                  rendaind < 789 ~ "at? 1 SM",
                                  rendaind %in% 789:1576 ~ "entre 1 e 2 SM",
                                  rendaind > 1576 ~ "mais de 2 SM",
                                  TRUE ~ NA_character_)),
                                Renda2 = factor(case_when(
                                  rendaind < 789 ~ "at? 1 SM",
                                  rendaind %in% 789:1576 ~ "entre 1 e 2 SM",
                                  rendaind %in% 1576:2364 ~ "entre 2 e 3 SM",
                                  rendaind %in% 2364:3152 ~ "entre 3 e 4 SM",
                                  rendaind > 3152 ~ "mais de 4 SM",
                                  TRUE ~ NA_character_)),
                                cirurgia_no_braco = factor(case_when(
                                  mf25 == 0 ~ "nao",
                                  mf25 == 1 ~ "sim",
                                  TRUE ~ NA_character_)),
                                medida_1 = factor(case_when(
                                  mf27 == 9555 ~ "tentou,mas /n conseguiu",
                                  mf27 == 9666 ~ "n tentou",
                                  mf27 == 9777 ~ "incapacitado",
                                  mf27 == 9888 | mf27 == 8888 ~ "recusou-se",
                                  mf27 < 100 ~ "realizou",
                                  TRUE ~ NA_character_)),
                                medida_2 = factor(case_when(
                                  mf28 == 9555 ~ "tentou,mas /n conseguiu",
                                  mf28 == 9666 ~ "n tentou",
                                  mf28 == 9777 ~ "incapacitado",
                                  mf28 == 9888 | mf28 == 8888 ~ "recusou-se",
                                  mf28 < 100 ~ "realizou",
                                  TRUE ~ NA_character_)),
                                medida_3 = factor(case_when(
                                  mf29 == 9555 ~ "tentou,mas /n conseguiu",
                                  mf29 == 9666 ~ "n tentou",
                                  mf29 == 9777 ~ "incapacitado",
                                  mf29 == 9888 | mf29 == 8888 ~ "recusou-se",
                                  mf29 < 100 ~ "realizou",
                                  TRUE ~ NA_character_)),
```

```
                                  medida_AL1 = factor(case_when(
                                    mf11 == 99999 ~ "achou arriscado",
                                    mf11 == 99888 ~ "incapacitado",
                                    mf11 == 99777 ~ "acamado",
                                    mf11 == 99666 ~ "recusou-se",
                                    mf11 < 3 ~ "realizou",
                                    TRUE ~ NA_character_)),
                                  medida_PS1 = factor(case_when(
                                    mf20 == 99999 ~ "achou arriscado",
                                    mf20 == 99888 ~ "incapacitado",
                                    mf20 == 99777 ~ "acamado",
                                    mf20 == 99666 ~ "recusou-se",
                                    mf20 < 200 ~ "realizou",
                                    TRUE ~ NA_character_)),
                                  elegivel_altura = factor(case_when(
                                    mf13 < 3 ~ "sim",
                                    mf13 > 3 ~ "não",
                                    TRUE ~ NA_character_)),
                                  elegivel_peso = factor(case_when(
                                    mf22 < 200 ~ "sim",
                                    mf22 > 200 ~ "não",
                                    TRUE ~ NA_character_)),
                                  elegivel_forca = factor(case_when(     #
strength in 5-70 kgf
                                    mf29 %in% 5:70 ~ "sim",
                                    mf29 > 70 | mf29 < 5 ~ "não",
                                    TRUE ~ NA_character_)),
                                  elegivel_forca2 = factor(case_when(    #any
strength under 75 kgf
                                    mf29 < 75 ~ "sim",
                                    mf29 > 75 | mf29 == 75 ~ "não",
                                    TRUE ~ NA_character_)))


  ########################################
  # Analyzing the age information quality
  ########################################

  # Objective: assess the quality of age information by checking whether the
questionnaire variables
  # (E0 = age at interview in completed years; nasc = date of birth) are
available in the dataset
  # (ELSI_2015). If present, these variables may have been used to construct
the released variable
  # "idade", potentially improving data quality by reconciling the declared
age (E0) with date of birth (nasc).

  # --- Check whether E0 and nasc exist in the dataset ---
  vars_to_check <- c("E0", "nasc", "idade")

  # Quick TRUE/FALSE check
  vars_present <- setNames(vars_to_check %in% names(ELSI_15), vars_to_check)
  print(vars_present)

  # List any missing variables (if any)
  missing_vars <- setdiff(vars_to_check, names(ELSI_15))
  if (length(missing_vars) == 0) {
    message("All target variables are present in ELSI_2015: ",
paste(vars_to_check, collapse = ", "))
  } else {
    message("Missing in ELSI_2015: ", paste(missing_vars, collapse = ", "))
  }
```

```
0541
0542  ##########################################
0543  # Age pyramid (single-year ages) by sex
0544  # Denominator: total weighted sample (men + women), so bars show the %
distribution by single-year age.
0545  # Fix: youngest ages at the bottom (base) and oldest at the top.
0546  ##########################################
0547
0548  Variables <- Variables_2015
0549
0550  # 1) Restrict to valid single-year ages and non-missing sex
0551  Vars_age <- Variables %>%
0552    filter(!is.na(idade), !is.na(Sexo), idade >= 50, idade <= 110) %>%
0553    mutate(
0554      idade_simple = as.integer(idade),
0555      one = 1
0556    )
0557
0558  # 2) Weighted totals by age and sex (numerators)
0559  age_sex_totals <- Vars_age %>%
0560    group_by(idade_simple, Sexo) %>%
0561    summarise(w_total = survey_total(one, na.rm = TRUE), .groups = "drop")
0562
0563  # 3) Overall weighted total (denominator: total sample)
0564  overall_total <- Vars_age %>%
0565    summarise(w_total_all = survey_total(one, na.rm = TRUE)) %>%
0566    dplyr::pull(w_total_all)
0567
0568  # 4) Convert to percentages of the total sample and build signed values
for pyramid
0569  age_pyramid_df <- age_sex_totals %>%
0570    mutate(
0571      pct = 100 * (w_total / overall_total),
0572      pct_signed = ifelse(Sexo == "masculino", -pct, pct),
0573      # youngest at bottom, oldest at top
0574      idade_simple_f = factor(idade_simple, levels =
sort(unique(idade_simple)))
0575    )
0576
0577  # 5) Plot
0578  p_age_pyramid <- ggplot(age_pyramid_df, aes(x = pct_signed, y =
idade_simple_f, fill = Sexo)) +
0579    geom_col(width = 0.9) +
0580    labs(
0581      x = "Percent of total sample (weighted, %)",
0582      y = "Age (single-year)",
0583      title = "Survey-weighted age pyramid (single-year ages) by sex",
0584      fill = "Sex"
0585    ) +
0586    scale_x_continuous(labels = function(x) paste0(abs(x), "%")) +
0587    theme_minimal()
0588
0589  print(p_age_pyramid)
0590
0591  ##########################################
0592  # Unweighted age pyramid (single-year ages) by sex
0593  # Purpose: replicate the age pyramid ignoring the complex survey design
(no weights).
0594  # Denominator: total unweighted sample (men + women), so bars show the %
distribution by single-year age.
0595  ##########################################
0596
```

```r
# Use the raw data frame (no survey design)
df <- ELSI_15

# 1) Keep valid ages and non-missing sex (adjust age range if needed)
df_age <- df %>%
  filter(!is.na(idade), !is.na(sexo), idade >= 50, idade <= 110) %>%
  mutate(
    idade_simple = as.integer(idade),
    Sexo = factor(ifelse(sexo == 1, "masculino", "feminino"),
                  levels = c("masculino", "feminino"))
  )

# 2) Unweighted counts by age and sex
age_sex_counts <- df_age %>%
  count(idade_simple, Sexo, name = "n")

# 3) Denominator: total unweighted sample size
N_total <- nrow(df_age)

# 4) Convert to % of total sample and build signed values for pyramid
age_pyramid_unw <- age_sex_counts %>%
  mutate(
    pct = 100 * (n / N_total),
    pct_signed = ifelse(Sexo == "masculino", -pct, pct),
    # youngest at bottom, oldest at top
    idade_simple_f = factor(idade_simple, levels =
sort(unique(idade_simple)))
  )

# 5) Plot
p_age_pyramid_unw <- ggplot(age_pyramid_unw, aes(x = pct_signed, y =
idade_simple_f, fill = Sexo)) +
  geom_col(width = 0.9) +
  labs(
    x = "Percent of total sample (unweighted, %)",
    y = "Age (single-year)",
    title = "Unweighted age pyramid (single-year ages) by sex",
    fill = "Sex"
  ) +
  scale_x_continuous(labels = function(x) paste0(abs(x), "%")) +
  theme_minimal()

print(p_age_pyramid_unw)

# Quality check (age reporting): Visual inspection of the single-year age
distributions (weighted and unweighted)
# did not suggest pronounced digit preference (e.g., heaping at ages
ending in 0 or 5). In our workflow,
# single-year ages are used in the intermediate step to compute age-
specific median handgrip strength and
# estimate sex-specific linear regressions at the national level;
quinquennial age groups are used only in the
# final step, when computing relative ages based on the age-group median
handgrip strength.


##########################################
# Age heaping checks (Whipple, Myers, digit histogram)
# Requirements: single-year, weighted counts for ages 50+.
# Notes:
# - Classic Whipple uses 25–60, but your dataset starts at 50. We compute
#   a main window 50–80 (broader, more stable) and provide sensitivity for
```

```
#   50–70 and 50–60 (narrower, closer to the classic upper bound).
# - Myers can run on any span; we'll use 50–95 by default (adjustable).
##########################################

library(DemoTools)

# Min e máx ages
ELSI_15 %>%
  dplyr::summarise(min_age = min(idade, na.rm = TRUE),
                   max_age = max(idade, na.rm = TRUE))

ELSI_15 %>%
  dplyr::filter(!is.na(idade), idade >= 100) %>%
  dplyr::count(idade) %>%
  dplyr::arrange(desc(idade))

# Ages 100+: we observe 1 case at age 104 and 3 at age 105.
# Because age 104 is present, this does not look like a "105+" top-code.
# Given the very small counts at extreme ages, we keep the analysis
windows
# focused to limit tail influence (Whipple: 50–80; Myers: 50–95).

# 0) Define analysis spans
age_min_all   <- 50      # min
age_max_all   <- 105     # max

# Main/reporting spans
whipple_main  <- c(50, 80)  # broader and stabler within your 50+ data
whipple_sens1 <- c(50, 70)  # sensitivity 1
whipple_sens2 <- c(50, 60)  # sensitivity 2 (closest to classic)
myers_span    <- c(50, 95)  # typical broad span for Myers

# 1) Build weighted single-year counts (50..age_max_all)
# We aggregate with the declared survey design (srvyr), then coerce to a
plain vector.
ages_full <- age_min_all:age_max_all

# I build a sequential integer vector from 50 to 105 based on the limits I
set above.
# I will use it in a right_join() to force every single-year age (50..105)
to appear
# in the final table—even if some ages are absent in the data; those will
be zero-filled.
# This avoids gaps in the age series and makes the Whipple/Myers
computations easier and safer.

age_counts_w <- Variables_2015 %>%
  filter(!is.na(idade), idade >= age_min_all, idade <= age_max_all) %>% #
Filters the valid universe: drops NA in idade and keeps only 50..105 (using
age_min_all/age_max_all).
  group_by(idade) %>%
#Aggregates by single-year age using the declared survey design (Variables_2015
from srvyr).
  summarise(n_w = survey_total(1, na.rm = TRUE), .groups = "drop") %>%  #
survey_total(1) computes the survey-weighted total of persons at each age
(variance not needed here).
                # ensure a full, gapless vector (zero-fill absent ages)
  right_join(tibble(idade = ages_full), by = "idade") %>%
#Performs a right_join to the complete age support ages_full (50..105) to force
every age to be present.
  mutate(n_w = ifelse(is.na(n_w), 0, as.numeric(n_w))) %>%             #
Zero-fills missing counts (turns NA into 0) and coerces n_w to plain numeric.
```

```
  arrange(idade)

age_counts_w

Age <- age_counts_w$idade
Pop <- age_counts_w$n_w

length(Age) == length(Pop)  # should be TRUE
any(is.na(Age))             # should be FALSE
any(is.na(Pop))             # should be FALSE (zeros ok)



# 2) Whipple index (main + sensitivities)
# Whipple in DemoTools returns values on a 1–5 scale. The UN "classic" 100–500
# scale is obtained by multiplying by 100. Interpretation bands were defined
# for 23–62 or 25–60; with 50+ data, treat thresholds as indicative.

W_main  <- check_heaping_whipple(Pop, Age,
                                 ageMin = whipple_main[1],
                                 ageMax = whipple_main[2],
                                 digit  = c(0, 5))
W_sens1 <- check_heaping_whipple(Pop, Age,
                                 ageMin = whipple_sens1[1],
                                 ageMax = whipple_sens1[2],
                                 digit  = c(0, 5))
W_sens2 <- check_heaping_whipple(Pop, Age,
                                 ageMin = whipple_sens2[1],
                                 ageMax = whipple_sens2[2],
                                 digit  = c(0, 5))

W_main_UN  <- 100 * W_main
W_sens1_UN <- 100 * W_sens1
W_sens2_UN <- 100 * W_sens2

# Whipple index for ages 50–80 (main) and two sensitivity windows (50–70, 50–60),
# focusing on digit preference at 0/5. DemoTools returns 1–5; we convert to the
# UN 100–500 scale for reporting (≈100 = no heaping). Consistent values across
# windows support robustness within a 50+ universe.

W_main_UN
W_sens1_UN
W_sens2_UN

# Age heaping was minimal. Whipple’s index (UN 100–500 scale) was 104.6 for ages 50–80,
# with close sensitivity results for 50–70 (104.9) and 50–60 (104.9),
# indicating negligible preference for digits 0/5 in our 50+ sample.

#################################################################
# 3) Myers blended index (total + by-digit percentages)
#################################################################

# Returns: total (0..90; lower is better) and a 10-element vector with
# terminal-digit shares (should be ~10% each in high-quality data).

M_total <- check_heaping_myers(Pop, Age,
```

```
                                  ageMin = myers_span[1],
                                  ageMax = myers_span[2])

M_total

# Compute terminal-digit shares manually within the Myers window (50–95)
a0 <- myers_span[1]; a1 <- myers_span[2]
sel <- Age >= a0 & Age <= a1

# weighted shares by terminal digit 0..9
digit_df <- dplyr::tibble(digit = Age[sel] %% 10, w = Pop[sel]) %>%
  dplyr::group_by(digit) %>%
  dplyr::summarise(n = sum(w), .groups = "drop") %>%
  # ensure all digits 0..9 are present (fill missing with 0)
  dplyr::right_join(dplyr::tibble(digit = 0:9), by = "digit") %>%
  dplyr::mutate(n = ifelse(is.na(n), 0, n),
                percent = 100 * n / sum(n)) %>%
  dplyr::arrange(digit)

digit_df        # coluna 'percent' deve ficar ~10% para cada dígito em dados de boa qualidade
round(digit_df$percent, 2)
sum(digit_df$percent)  # ~100

digit_df

# --- Manuscript (Results) ---
# Age reporting quality was high. Whipple’s index for ages 50–80 (UN 100–500 scale)
# was 104.6 (sensitivities: 50–70 = 104.9; 50–60 = 104.9), indicating negligible
# heaping at digits 0/5. Myers’ blended index for ages 50–95 was 2.67 (0–90 scale;
# lower is better), consistent with minimal overall digit preference.

# --- Supplementary (Figure / Interpretation) ---
# Terminal-digit shares (ages 50–95) were approximately uniform (~10% each), with a
# mild tilt toward digits 0–4 (≈10.5–11.3%) and slightly lower shares for 5–9
# (≈8.0–9.8%). There were no isolated spikes at 0 or 5.

# --- Methods (brief, to cite windows and scales) ---
# We assessed age heaping using Whipple’s index focused on ages 50–80 and on two
# sensitivity windows (50–70; 50–60). Results are reported on the UN 100–500 scale
# (≈100 indicates no heaping). We also computed Myers’ blended index over ages 50–95
# (0–90 scale; lower values indicate less digit preference). Choice of windows reflects
# the study universe (50+) and aims to reduce tail influence while preserving comparability.


ggplot2::ggplot(digit_df, ggplot2::aes(x = factor(digit), y = percent)) +
  ggplot2::geom_col() +
  ggplot2::geom_hline(yintercept = 10, linetype = 2) +
  ggplot2::labs(title = "Terminal digit distribution of ages (weighted, 50–95)",
                x = "Terminal digit (0–9)", y = "Percent") +
  ggplot2::theme_minimal()
```

```
0805
0806  # Interpretation of the terminal-digit bar chart (ages 50–95):
0807  # Bars hover near the 10% reference line, with a mild tilt toward digits
0–4
0808  # and slightly lower shares for digits 5–9. There are no isolated spikes
at 0 or 5.
0809  # This visual pattern aligns with a low Myers index (2.67) and Whipple
≈105,
0810  # supporting the conclusion of minimal age heaping.
0811
0812  # Suggested figure caption (manuscript/supplement):
0813  # "Terminal-digit distribution of single-year ages (weighted, ages 50–95).

0814  # Shares are approximately uniform (~10% each), with a mild tilt toward
digits 0–4
0815  # and no isolated spikes at 0 or 5, consistent with low age heaping."
0816
0817
0818
0819  # 4) Compact summary table for reporting
0820  heaping_summary <- tibble(
0821    index               = c("Whipple (50–80)", "Whipple (50–70)", "Whipple
(50–60)", "Myers (50–95)"),
0822    value               = c(W_main_UN, W_sens1_UN, W_sens2_UN, M_total),
0823    scale               = c("100–500", "100–500", "100–500", "0–90"),
0824    interpretation_note = c(
0825      "UN scale; thresholds are indicative here (50+ span). Lower ~ better;
100≈no heaping.",
0826      "UN scale; sensitivity window.",
0827      "UN scale; closest to classic upper bound; treat cautiously (narrow
span).",
0828      "Lower ~ better; 0≈no preference; also inspect digit histogram."
0829    )
0830  )
0831
0832  print(heaping_summary)
0833
0834  # Age reporting quality was high. Whipple’s index on ages 50–80 (UN 100–
500 scale)
0835  # was ~105, with consistent sensitivity checks for 50–70 and 50–60,
indicating
0836  # negligible heaping at digits 0/5. Myers’ blended index on ages 50–95 was
2.67
0837  # (0–90 scale; lower is better), consistent with minimal overall digit
preference.
0838
0839
0840
################################################################################
0841  ## Exploratory stage: defining eligible observations for the study.
0842
################################################################################
0843
0844  # An individual is considered eligible if they meet ALL of the following:
0845  # 1) Has NOT undergone arm surgery
0846  # 2) Completed all three grip-strength measures
0847  # 3) Has a recorded body weight measurement
0848  # 4) Has a recorded body height measurement
0849
0850  ################################################
0851  # grip-strength measures
0852  ################################################
```

```
0853
0854  # Weighted cross-tabs and column percentages by sex (exclude NAs from
denominators)
0855
0856  # First measure (mf27)
0857  d1 <- subset(Variables_2015, !is.na(Sexo) & !is.na(medida_1))  # exclude
NAs to avoid distorted denominators
0858  tab2 <- svytable(~medida_1 + Sexo, design = d1)
0859  tab2
0860  prop.table(tab2, margin = 2) * 100  # column percentages (by sex)
0861
0862  # Second measure (mf28)
0863  d2 <- subset(Variables_2015, !is.na(Sexo) & !is.na(medida_2))
0864  tab3 <- svytable(~medida_2 + Sexo, design = d2)
0865  tab3
0866  prop.table(tab3, margin = 2) * 100  # column percentages (by sex)
0867
0868  # Third measure (mf29)
0869  d3 <- subset(Variables_2015, !is.na(Sexo) & !is.na(medida_3))
0870  tab4 <- svytable(~medida_3 + Sexo, design = d3)
0871  tab4
0872  prop.table(tab4, margin = 2) * 100  # column percentages (by sex)
0873
0874  rm(tab2,tab3,tab4)
0875
0876  ELSI_2015_sub <- dplyr::select(ELSI_15, id, mf27, mf28, mf29)
0877
0878  # NOTE ON WEIGHTED “COUNTS” FROM svytable():
0879  # The totals produced by svytable() are sums of the survey weights within
each cell, i.e., weighted totals.
0880  # In our output for the first handgrip trial (mf27, mf28, mf29), these
totals are close to the sample size (and appear
0881  # with decimals), rather than “exploding” to national population
magnitudes. This pattern is consistent with
0882  # weights that are calibrated/normalized to an analytic (sample) scale—
where sum(weights) ≈ N—rather than
0883  # expansion weights—where sum(weights) ≈ target population size.
0884  #
0885  # Under normalized/calibrated weights, percentages, means, and regression
coefficients remain valid for
0886  # design-based inference (multiplying all weights by a constant does not
change ratios/means), but the raw
0887  # weighted totals from svytable() should not be interpreted as the number
of people in Brazil. Instead, they
0888  # represent weighted totals on the design’s scale. If population-level
totals are required, weights must be
0889  # re-scaled using an external benchmark for the target population (e.g.,
total population 50+ in the
0890  # corresponding year), and then totals should be recomputed under that re-
scaled design.
0891
0892
0893  ################################################
0894  # variables related to weight and height
0895  ################################################
0896
0897  # Height
0898  tab44 <- svytable(~medida_AL1+Sexo, design=Variables_2015)
0899  tab44
0900  proportions(tab44, margin = 2)*100
0901
0902  rm(tab44)
```

```r
# Weight
tab45 <- svytable(~medida_PS1+Sexo, design=Variables_2015)
tab45
proportions(tab45, margin = 2)*100

rm(tab44,tab45)



################################################
# Selected
################################################

#Height
tab46 <- svytable(~elegivel_altura+Sexo, design=Variables_2015)
tab46
proportions(tab46, margin = 2)*100

# Weight
tab47 <- svytable(~elegivel_peso+Sexo, design=Variables_2015)
tab47
proportions(tab47, margin = 2)*100

#strenght
tab48<- svytable(~elegivel_forca+Sexo, design=Variables_2015)
tab48
proportions(tab48, margin = 2)*100

rm(tab46,tab47,tab48)



#####################################################
# Analyzing the three handgrip strength measurements
#####################################################

summary(ELSI_15$mf29)

mf27_clean <- ELSI_15$mf27[ELSI_15$mf27 < 200]
summary(mf27_clean)

mf28_clean <- ELSI_15$mf28[ELSI_15$mf28 < 200]
summary(mf28_clean)

mf29_clean <- ELSI_15$mf29[ELSI_15$mf29 < 200]
summary(mf29_clean)



#########################################################################
# first option - individuals with any measure of strength in 5-70 kgf
#########################################################################


# Keep only individuals with complete handgrip (3 trials),
# non-missing weight and height, and measured grip strength in 5-70 kgf.


Selected_data <- Variables_2015 %>% filter (elegivel_altura == "sim",
                                            elegivel_peso == "sim",
                                            elegivel_forca == "sim")

```

```
Number <- as.data.frame(Selected_data)
# 8,675 individuals remain in the sample with this new filter
rm(Number)



########################################################################
# Second option - individuals with any measure of strength under 75 kgf
########################################################################

# Keep only individuals with complete handgrip (3 trials),
# non-missing weight and height, and measured grip strength under 70 kgf.

Selected_data2 <- Variables_2015 %>% filter (elegivel_altura == "sim",
                                        elegivel_peso == "sim",
                                        elegivel_forca2 == "sim")

Number2 <- as.data.frame(Selected_data2)
# 8694 individuals remain in the sample with this new filter



########################################################################
# analyzing who was excluded from the sample because grip strength is not between 5 and 70 kgf
########################################################################

# identify the 19 observations that are included in Option 2 but excluded in Option 1 (unweighted sample)

# convert to data.frame and create a stable row index for set operations
df_vars2015 <- Variables_2015 %>%
  as.data.frame() %>%
  mutate(.rowid = dplyr::row_number())

# Option 1 sample (5–70 kgf rule)
opt1 <- df_vars2015 %>%
  filter(
    elegivel_altura == "sim",
    elegivel_peso   == "sim",
    elegivel_forca  == "sim"
  ) %>%
  select(.rowid, Sexo, idade, Regiao, elegivel_forca, elegivel_forca2, mf29)

# Option 2 sample (< 75 kgf rule)
opt2 <- df_vars2015 %>%
  filter(
    elegivel_altura == "sim",
    elegivel_peso   == "sim",
    elegivel_forca2 == "sim"
  ) %>%
  select(.rowid, Sexo, idade, Regiao, elegivel_forca, elegivel_forca2, mf29)

# the 19 additional cases: in Option 2 but not in Option 1
diff_2_not_1 <- opt2 %>%
  anti_join(opt1, by = ".rowid")

# quick checks
nrow(opt1)
```

```r
nrow(opt2)
nrow(diff_2_not_1)

# 1) Sexo x idade (unweighted counts)
tab_sexo_idade <- diff_2_not_1 %>%
  count(Sexo, idade, name = "n") %>%
  arrange(Sexo, idade)

# 2) Sexo x Região (unweighted counts)
tab_sexo_regiao <- diff_2_not_1 %>%
  count(Sexo,Regiao, name = "n") %>%
  arrange(Sexo, Regiao)

# display in the console
tab_sexo_idade
tab_sexo_regiao

# create a table with the 19 additional cases (Option 2 but not Option 1) and export to Excel

# convert to data.frame and create a stable row index for set operations
df_vars2015 <- Variables_2015 %>%
  as.data.frame() %>%
  mutate(.rowid = dplyr::row_number())

# Option 1 sample (5–70 kgf rule)
opt1 <- df_vars2015 %>%
  filter(
    elegivel_altura == "sim",
    elegivel_peso   == "sim",
    elegivel_forca  == "sim"
  ) %>%
  select(.rowid)

# Option 2 sample (< 75 kgf rule)
opt2 <- df_vars2015 %>%
  filter(
    elegivel_altura == "sim",
    elegivel_peso   == "sim",
    elegivel_forca2 == "sim"
  )

# the 19 additional cases: in Option 2 but not in Option 1
diff_2_not_1 <- opt2 %>%
  anti_join(opt1, by = ".rowid")

# build the requested table
tab_19 <- diff_2_not_1 %>%
  transmute(
    id      = id,
    idade   = idade,
    Sexo    = as.character(Sexo),
    Regiao  = as.character(Regiao),
    mf27    = mf27,
    mf28    = mf28,
    mf29    = mf29
  )

# export to Excel (writexl)
xlsx_file <- "excluded_19_individuals.xlsx"
writexl::write_xlsx(tab_19, path = xlsx_file)
```

```
# confirm path
normalizePath(xlsx_file)



#####################################################################
# calculating the body mass index (BMI)
#####################################################################

Selected_data <- Selected_data %>% mutate(IMC = mf22/(mf13*mf13))


# filtering BMI below 18.5
removidos <- Selected_data %>% filter (IMC<18.5)
baixo_peso <- as.data.frame(removidos) # 186 underweight individuals were removed
rm(removidos)

# filtering BMI above 50
removidos <- Selected_data %>% filter (IMC>50)
obesidade_morbida <- as.data.frame(removidos) # 7 individuals with morbid obesity were removed
rm(removidos)


#####################################################
##### NEW DATABASE and STRENGTH MEASURE ############
#####################################################

Eligible_dataset <- Selected_data %>% filter (IMC>18.4,IMC<51)
Dataset <- as.data.frame(Eligible_dataset)
# at this point, 8,494 individuals remain in the sample
rm(Dataset)

#####################################################
##### Calculating the sample mean age #############
#####################################################

res <- Eligible_dataset %>%
  summarise(
    mean_age = survey_mean(idade, na.rm = TRUE),
    sd_age   = survey_sd(idade, na.rm = TRUE)
  )

cat("Mean age:", round(res$mean_age, 1), "+/-", round(res$sd_age, 1), "\n")


#################################################
# calculating mean handgrip strength
Eligible_dataset <- Eligible_dataset %>% mutate(GRIP_STRENGTH = (mf27+mf28+mf29)/3)

# calculating a test variable (GRIP_STRENGTH/IMC)
# and removing two more observations with strength lower than 5kg
Eligible_dataset <- Eligible_dataset %>% filter (GRIP_STRENGTH > 4.9) %>%
  mutate(TEST_VAR = GRIP_STRENGTH/IMC)
Number <- as.data.frame(Eligible_dataset)
nrow(Number)
# at this point, 8492 individuals remain in the sample
rm(Number)

```

```r
##################################################
###### NEW TABLE 1, only with eligible individuals
##################################################

##############################################
# Table by sex + export to Excel
# Requires: existing objects 'Valid_measure', 'Sexo' and 'IMC'
##############################################

# packages
suppressPackageStartupMessages({
  library(dplyr)
  library(survey)
  library(srvyr)
})

# 1) Build the table --------
# n = unweighted; mean/SD = weighted by the sample design
tab1_sex <- Eligible_dataset %>%
  group_by(Sexo) %>%
  summarise(
    n = unweighted(n()),                          # raw count after exclusions
    media_imc = survey_mean(IMC, na.rm = TRUE),  # weighted mean
    var_imc   = survey_var(IMC,  na.rm = TRUE)   # weighted variance (design-based)
  ) %>%
  mutate(
    dp_imc    = sqrt(var_imc)                  # weighted SD
  ) %>%
  # keep everything "clean" for export
  transmute(
    Sexo = as.character(Sexo),
    `n (não ponderado)`        = as.integer(n),
    `IMC – média (ponderada)`  = round(as.numeric(media_imc), 2),
    `IMC – DP (ponderado)`     = round(as.numeric(dp_imc), 2)
  ) %>%
  arrange(factor(Sexo, levels = c("masculino", "feminino")))

# display in the console
print(tab1_sex)

# 2) Export to Excel --------
# Attempt 1: writexl; Attempt 2: openxlsx; fallback: CSV
arquivo_xlsx <- "Tabela1_por_sexo.xlsx"

tryCatch({
  if (requireNamespace("writexl", quietly = TRUE)) {
    writexl::write_xlsx(list("Tabela1_por_sexo" = tab1_sex), path = arquivo_xlsx)
  } else if (requireNamespace("openxlsx", quietly = TRUE)) {
    openxlsx::write.xlsx(tab1_sex, file = arquivo_xlsx, sheetName = "Tabela1_por_sexo", overwrite = TRUE)
  } else {
    stop("Sem writexl e openxlsx instalados")
  }
  message("✔ Arquivo Excel salvo em: ", normalizePath(arquivo_xlsx))
}, error = function(e) {
  warning("Falha ao salvar Excel (", e$message, "). Salvando CSV como alternativa.")
  utils::write.csv(tab1_sex, file = sub("\\.xlsx$", ".csv", arquivo_xlsx), row.names = FALSE)
```

```
1196    message("✔ Arquivo CSV salvo em: ", normalizePath(sub("\\.xlsx$",
".csv", arquivo_xlsx)))
1197  })
1198
1199  ####################################
1200  # second part of Table 1
1201  ###### Idade2 ######################
1202
1203  ############################################
1204  # Table by Idade2 + export to Excel
1205  # Requires: object 'eligible_dataset' (srvyr/survey) with Idade2 and IMC
1206  ############################################
1207
1208  # 1) Build the table ---
1209  # n = unweighted; mean/SD = weighted (design-based)
1210  tab_idade2 <- Eligible_dataset %>%
1211    filter(!is.na(Idade2)) %>%
1212    group_by(Idade2) %>%
1213    summarise(
1214      n         = unweighted(n()),                # raw count after
exclusions
1215      media_imc = survey_mean(IMC, na.rm = TRUE),  # weighted mean
1216      var_imc   = survey_var(IMC,  na.rm = TRUE)   # weighted variance
1217    ) %>%
1218    mutate(dp_imc = sqrt(var_imc)) %>%            # weighted SD
1219    arrange(Idade2) %>%                           # preserves Idade2 factor
order
1220    transmute(
1221      `Faixa etária (Idade2)`   = as.character(Idade2),
1222      `n (não ponderado)`        = as.integer(n),
1223      `IMC – média (ponderada)`  = round(as.numeric(media_imc), 2),
1224      `IMC – DP (ponderado)`     = round(as.numeric(dp_imc), 2)
1225    )
1226
1227  # display in the console
1228  print(tab_idade2)
1229
1230  # 2) Export to Excel (with CSV fallback) ---
1231  arquivo_xlsx <- "Tabela1_por_Idade2.xlsx"
1232
1233  tryCatch({
1234    if (requireNamespace("writexl", quietly = TRUE)) {
1235      writexl::write_xlsx(list("Tabela1_por_Idade2" = tab_idade2), path =
arquivo_xlsx)
1236    } else if (requireNamespace("openxlsx", quietly = TRUE)) {
1237      openxlsx::write.xlsx(tab_idade2, file = arquivo_xlsx,
1238                           sheetName = "Tabela1_por_Idade2", overwrite =
TRUE)
1239    } else {
1240      stop("Sem writexl e openxlsx instalados")
1241    }
1242    message("✔ Arquivo Excel salvo em: ", normalizePath(arquivo_xlsx))
1243  }, error = function(e) {
1244    warning("Falha ao salvar Excel (", e$message, "). Salvando CSV como
alternativa.")
1245    utils::write.csv(tab_idade2,
1246                     file = sub("\\.xlsx$", ".csv", arquivo_xlsx),
1247                     row.names = FALSE)
1248    message("✔ Arquivo CSV salvo em: ",
1249            normalizePath(sub("\\.xlsx$", ".csv", arquivo_xlsx)))
1250  })
1251
```

```
1252
1253  ######################################
1254  #### Third part of the table: region
1255  ######################################
1256
1257  ##########################################
1258  # Table by Region + export to Excel
1259  # Requires: object 'Eligeble_dataset' (srvyr/survey) with 'regiao' and
'IMC'
1260  ##########################################
1261
1262  # 1) Build the table
1263  # n = unweighted; mean/SD = weighted (design-based)
1264  tab_regiao <- Eligible_dataset %>%
1265    filter(!is.na(regiao)) %>%
1266    group_by(regiao) %>%
1267    summarise(
1268      n         = unweighted(n()),                # raw count after
exclusions
1269      media_imc = survey_mean(IMC, na.rm = TRUE),  # weighted mean
1270      var_imc   = survey_var(IMC,  na.rm = TRUE)   # weighted variance
1271    ) %>%
1272    mutate(dp_imc = sqrt(var_imc)) %>%            # weighted SD
1273    arrange(regiao) %>%                           # preserves the existing
factor order
1274    transmute(
1275      `Região`                    = as.character(regiao),
1276      `n (não ponderado)`         = as.integer(n),
1277      `IMC – média (ponderada)`   = round(as.numeric(media_imc), 2),
1278      `IMC – DP (ponderado)`      = round(as.numeric(dp_imc), 2)
1279    )
1280
1281  # display in the console
1282  print(tab_regiao)
1283
1284  # --- 2) Export to Excel (with CSV fallback) ---
1285  arquivo_xlsx <- "Tabela1_por_Regiao.xlsx"
1286
1287  tryCatch({
1288    if (requireNamespace("writexl", quietly = TRUE)) {
1289      writexl::write_xlsx(list("Tabela1_por_Regiao" = tab_regiao), path =
arquivo_xlsx)
1290    } else if (requireNamespace("openxlsx", quietly = TRUE)) {
1291      openxlsx::write.xlsx(tab_regiao, file = arquivo_xlsx,
1292                           sheetName = "Tabela1_por_Regiao", overwrite =
TRUE)
1293    } else {
1294      stop("Sem writexl e openxlsx instalados")
1295    }
1296    message("✔ Arquivo Excel salvo em: ", normalizePath(arquivo_xlsx))
1297  }, error = function(e) {
1298    warning("Falha ao salvar Excel (", e$message, "). Salvando CSV como
alternativa.")
1299    utils::write.csv(tab_regiao,
1300                     file = sub("\\.xlsx$", ".csv", arquivo_xlsx),
1301                     row.names = FALSE)
1302    message("✔ Arquivo CSV salvo em: ",
1303            normalizePath(sub("\\.xlsx$", ".csv", arquivo_xlsx)))
1304  })
1305
1306  ##########################################################
1307  # Table 4
```

```
1308  ##########################################################
1309
1310  # Unweighted cross-tab: counts (n) by Sexo (rows) and Regiao (columns)
1311  # (Uses Eligible_dataset, but converts to data.frame to ignore survey
weights.)
1312
1313  df_elig <- Eligible_dataset %>% as.data.frame()
1314
1315  tab_sexo_regiao <- df_elig %>%
1316    mutate(
1317      Sexo   = factor(as.character(Sexo), levels = c("masculino",
"feminino")),
1318      Regiao = factor(as.character(Regiao),
1319                      levels = c("Sudeste", "Nordeste", "Sul", "Centro-
Oeste", "Norte"))
1320    ) %>%
1321    count(Sexo, Regiao, name = "n") %>%
1322    tidyr::pivot_wider(
1323      names_from  = Regiao,
1324      values_from = n,
1325      values_fill = 0
1326    ) %>%
1327    arrange(Sexo)
1328
1329  tab_sexo_regiao
1330
1331  # Unweighted cross-tab: counts (n) by Idade2 (rows) and Regiao (columns)
1332  # (Uses Eligible_dataset, but converts to data.frame to ignore survey
weights.)
1333
1334  df_elig <- Eligible_dataset %>% as.data.frame()
1335
1336  tab_idade2_regiao <- df_elig %>%
1337    mutate(
1338      Idade2 = as.character(Idade2),
1339      Regiao = factor(as.character(Regiao),
1340                      levels = c("Sudeste", "Nordeste", "Sul", "Centro-
Oeste", "Norte"))
1341    ) %>%
1342    count(Idade2, Regiao, name = "n") %>%
1343    tidyr::pivot_wider(
1344      names_from  = Regiao,
1345      values_from = n,
1346      values_fill = 0
1347    ) %>%
1348    arrange(Idade2)
1349
1350  tab_idade2_regiao
1351
1352  # Unweighted cross-tab: counts (n) by Zona (rows) and Regiao (columns)
1353  # (Uses Eligible_dataset, but converts to data.frame to ignore survey
weights.)
1354
1355  df_elig <- Eligible_dataset %>% as.data.frame()
1356
1357  tab_zona_regiao <- df_elig %>%
1358    mutate(
1359      Zona  = factor(as.character(Zona), levels = c("urbana", "rural")),
1360      Regiao = factor(as.character(Regiao),
1361                      levels = c("Sudeste", "Nordeste", "Sul", "Centro-
Oeste", "Norte"))
1362    ) %>%
```

```
1363    count(Zona, Regiao, name = "n") %>%
1364    tidyr::pivot_wider(
1365      names_from  = Regiao,
1366      values_from = n,
1367      values_fill = 0
1368    ) %>%
1369    arrange(Zona)
1370
1371  tab_zona_regiao
1372
1373  # Unweighted cross-tab: counts (n) by Escolaridade (rows) and Regiao
(columns)
1374  # (Uses Eligible_dataset, but converts to data.frame to ignore survey
weights.)
1375
1376  df_elig <- Eligible_dataset %>% as.data.frame()
1377
1378  tab_escolaridade_regiao <- df_elig %>%
1379    mutate(
1380      Escolaridade = as.character(Escolaridade),
1381      Regiao = factor(as.character(Regiao),
1382                      levels = c("Sudeste", "Nordeste", "Sul", "Centro-
Oeste", "Norte"))
1383    ) %>%
1384    count(Escolaridade, Regiao, name = "n") %>%
1385    tidyr::pivot_wider(
1386      names_from  = Regiao,
1387      values_from = n,
1388      values_fill = 0
1389    ) %>%
1390    arrange(Escolaridade)
1391
1392  tab_escolaridade_regiao
1393
1394  # Unweighted cross-tab: counts (n) by Raca_cor (rows) and Regiao (columns)
1395  # (Uses Eligible_dataset, but converts to data.frame to ignore survey
weights.)
1396
1397  df_elig <- Eligible_dataset %>% as.data.frame()
1398
1399  tab_raca_regiao <- df_elig %>%
1400    mutate(
1401      Raca_cor = as.character(Raca_cor),
1402      Regiao   = factor(as.character(Regiao),
1403                        levels = c("Sudeste", "Nordeste", "Sul", "Centro-
Oeste", "Norte"))
1404    ) %>%
1405    count(Raca_cor, Regiao, name = "n") %>%
1406    tidyr::pivot_wider(
1407      names_from  = Regiao,
1408      values_from = n,
1409      values_fill = 0
1410    ) %>%
1411    arrange(Raca_cor)
1412
1413  tab_raca_regiao
1414
1415
1416  # Unweighted cross-tab: counts (n) by Renda (rows) and Regiao (columns)
1417  # (Uses Eligible_dataset, but converts to data.frame to ignore survey
weights.)
1418
```

```r
df_elig <- Eligible_dataset %>% as.data.frame()

tab_renda_regiao <- df_elig %>%
  mutate(
    Renda  = as.character(Renda),
    Regiao = factor(as.character(Regiao),
                    levels = c("Sudeste", "Nordeste", "Sul", "Centro-Oeste", "Norte"))
  ) %>%
  count(Renda, Regiao, name = "n") %>%
  tidyr::pivot_wider(
    names_from  = Regiao,
    values_from = n,
    values_fill = 0
  ) %>%
  arrange(Renda)

tab_renda_regiao


# Export multiple cross-tabs to a single Excel file (one sheet per table)
# Requires the following objects to exist in your environment:
# tab_sexo_regiao, tab_idade2_regiao, tab_zona_regiao, tab_escolaridade_regiao,
# tab_raca_regiao, tab_renda_regiao

arquivo_xlsx <- "Crosstabs_by_Region.xlsx"

sheets_list <- list(
  "Sexo_x_Regiao"          = tab_sexo_regiao,
  "Idade2_x_Regiao"        = tab_idade2_regiao,
  "Zona_x_Regiao"          = tab_zona_regiao,
  "Escolaridade_x_Regiao"  = tab_escolaridade_regiao,
  "RacaCor_x_Regiao"       = tab_raca_regiao,
  "Renda_x_Regiao"         = tab_renda_regiao
)

tryCatch({
  if (requireNamespace("writexl", quietly = TRUE)) {
    writexl::write_xlsx(sheets_list, path = arquivo_xlsx)
  } else if (requireNamespace("openxlsx", quietly = TRUE)) {
    wb <- openxlsx::createWorkbook()
    for (nm in names(sheets_list)) {
      openxlsx::addWorksheet(wb, nm)
      openxlsx::writeData(wb, nm, sheets_list[[nm]])
    }
    openxlsx::saveWorkbook(wb, arquivo_xlsx, overwrite = TRUE)
  } else {
    stop("Neither writexl nor openxlsx is installed.")
  }
  message("✔ Excel file saved at: ", normalizePath(arquivo_xlsx))
}, error = function(e) {
  stop("Export failed: ", e$message)
})

############################################################################
# ==========================================================
# Exploratory analysis: age vs GRIP_STRENGTH (ages 50–85), by sex,
# accounting for ELSI complex sampling (PSU/strata/weights)
# ==========================================================
############################################################################
library(survey)
```

```
library(dplyr)
library(ggplot2)

# ------------------------------------------------------------
# 1) Build an explicit svydesign object from Eligible_dataset
#    (assumes upa, estrato, and peso_calibrado are present)
# ------------------------------------------------------------

eligible_df <- as.data.frame(Eligible_dataset)

des_elig <- svydesign(
  ids     = ~upa,
  strata  = ~estrato,
  weights = ~peso_calibrado,
  data    = eligible_df,
  nest    = TRUE
)

# Restrict to ages 50–85 and complete cases on variables of interest
des_50_85 <- subset(
  des_elig,
  idade >= 50 & idade <= 85 &
    !is.na(idade) & !is.na(GRIP_STRENGTH) & !is.na(sexo)
)

# Sex-specific designs (ELSI coding: sexo==1 men, sexo==0 women)
des_m <- subset(des_50_85, sexo == 1)
des_f <- subset(des_50_85, sexo == 0)

# ------------------------------------------------------------
# 2) Design-based Pearson correlation (via svyvar)
#    Note: this yields the correlation coefficient; a formal test
#    for linear trend is typically obtained via svyglm (optional).
# ------------------------------------------------------------

corr_from_svyvar <- function(des){
  V <- svyvar(~idade + GRIP_STRENGTH, design = des, na.rm = TRUE)
  cov_ag <- as.numeric(V["idade", "GRIP_STRENGTH"])
  var_a  <- as.numeric(V["idade", "idade"])
  var_g  <- as.numeric(V["GRIP_STRENGTH", "GRIP_STRENGTH"])
  cov_ag / sqrt(var_a * var_g)
}

cor_m <- corr_from_svyvar(des_m)
cor_f <- corr_from_svyvar(des_f)

cat("\nDesign-based Pearson correlation (ages 50–85):\n")
cat("Men   :", round(cor_m, 4), "\n")
cat("Women :", round(cor_f, 4), "\n")

# INTERPRETATION (Design-based correlation, ages 50–85):
# The weighted (design-based) Pearson correlation between chronological
age and handgrip strength
# is modest in magnitude for both sexes (Men: r = -0.386; Women: r = -
0.327). In a simple linear
# framework, this corresponds to r^2 ≈ 0.149 for men and r^2 ≈ 0.107 for
women, meaning that age
# alone accounts for only ~15% and ~11% of the variability in
GRIP_STRENGTH, respectively.
# Therefore, although the direction is clearly negative, the dispersion at
the individual level is
# substantial and a single straight-line model with age as the only
```

```
predictor would have limited
1536  # explanatory and predictive value. For this reason, subsequent steps
prioritize descriptive,
1537  # design-based age profiles and/or more flexible functional forms (e.g.,
splines) rather than
1538  # relying on a simple linear regression fitted to all individual
observations.
1539
1540  # ------------------------------------------------------------
1541  # 3) Scatterplots (raw points) by sex to visually assess dispersion
1542  #    and whether a linear trend is plausible
1543  # ------------------------------------------------------------
1544
1545  plot_df <- eligible_df %>%
1546    filter(
1547      idade >= 50, idade <= 85,
1548      !is.na(idade), !is.na(GRIP_STRENGTH), !is.na(sexo)
1549    ) %>%
1550    mutate(Sex = ifelse(sexo == 1, "Men", "Women"))
1551
1552  ggplot(plot_df, aes(x = idade, y = GRIP_STRENGTH)) +
1553    geom_point(alpha = 0.15) +
1554    facet_wrap(~Sex, ncol = 1) +
1555    labs(
1556      x = "Age (years)",
1557      y = "Handgrip strength (kgf)",
1558      title = "Scatterplot of GRIP_STRENGTH vs age (50–85), by sex",
1559      subtitle = "Raw observations (visual check of dispersion and
linearity)"
1560    )
1561
1562  # ------------------------------------------------------------
1563  # 4) Design-based mean GRIP_STRENGTH by single-year age (with 95% CI),
1564  #    by sex. This is often more informative than raw scatter alone
1565  #    when assessing the functional form under complex sampling.
1566  # ------------------------------------------------------------
1567
1568  mean_by_age_m <- svyby(
1569    ~GRIP_STRENGTH, ~idade, design = des_m,
1570    FUN = svymean, na.rm = TRUE, vartype = "ci"
1571  ) %>%
1572    as.data.frame() %>%
1573    mutate(Sex = "Men")
1574
1575  mean_by_age_f <- svyby(
1576    ~GRIP_STRENGTH, ~idade, design = des_f,
1577    FUN = svymean, na.rm = TRUE, vartype = "ci"
1578  ) %>%
1579    as.data.frame() %>%
1580    mutate(Sex = "Women")
1581
1582  mean_by_age <- bind_rows(mean_by_age_m, mean_by_age_f)
1583
1584  ggplot(mean_by_age, aes(x = idade, y = GRIP_STRENGTH)) +
1585    geom_ribbon(aes(ymin = ci_l, ymax = ci_u), alpha = 0.20) +
1586    geom_line(linewidth = 1) +
1587    facet_wrap(~Sex, ncol = 1) +
1588    labs(
1589      x = "Age (years)",
1590      y = "Mean GRIP_STRENGTH (kgf)",
1591      title = "Design-based mean GRIP_STRENGTH by age (50–85), with 95% CI",
1592      subtitle = "Computed with svyby(svymean) under the complex survey
```

```
design"
1593    )
1594
1595  # NOTE:
1596  # A design-based linear model (svyglm) could be fitted to formally test
the age slope,
1597  # but given the modest correlation and large individual-level dispersion,
we do not
1598  # proceed with a simple linear regression as the primary approach in this
analysis.
1599
1600
1601
1602  ##############################################
1603  #### Exploring the dependent variable GRIP_STRENGTH
1604  ##############################################
1605
1606  # subset for men in general
1607  homens <- Eligible_dataset %>% select (idade,sexo,GRIP_STRENGTH) %>%
1608    filter(sexo == 1,idade<86) %>%
1609    group_by(idade) %>%
1610    summarise(
1611      total = survey_mean(GRIP_STRENGTH, na.rm =TRUE, vartype = NULL)) %>%
1612    drop_na()
1613
1614  homens2 <- as.data.frame(homens)
1615  plot(homens2$idade,homens2$total)
1616
1617
1618  # subset for women in general
1619  mulheres <- Eligible_dataset %>% select (idade,sexo,GRIP_STRENGTH) %>%
1620    filter(sexo == 0,idade<81) %>%
1621    group_by(idade) %>%
1622    summarise(
1623      total = survey_mean(GRIP_STRENGTH, na.rm =TRUE, vartype = NULL)) %>%
1624    drop_na()
1625
1626  mulheres2 <- as.data.frame(mulheres)
1627  plot(mulheres2$idade,mulheres2$total)
1628
1629  lista_dados <- list("homens" = homens,
1630                      "mulheres" = mulheres)
1631
1632  write.xlsx(lista_dados, file="forca_brasil.xlsx")
1633
1634
1635  ################################################
1636  #### Creating new plots for Article about Region
1637  ################################################
1638
1639  library(survey)
1640  library(dplyr)
1641  library(ggplot2)
1642
1643  options(survey.lonely.psu = "adjust")
1644
1645  # If it does not exist yet:
1646  design_ok <- Eligible_dataset %>% as_survey_design()
1647
1648  # 1) Weighted median by Sexo x idade (no CI; no variances)
1649  tab_med <- svyby(
1650    ~ GRIP_STRENGTH, ~ Sexo + idade,
```

```
1651    design = Eligible_dataset,
1652    FUN = svyquantile,
1653    quantiles = 0.5,
1654    ci = FALSE,           # does not attempt CI → does not call qt()
1655    keep.names = FALSE,
1656    keep.var = FALSE,
1657    na.rm = TRUE
1658  )
1659  tab_med <- as.data.frame(tab_med)
1660
1661  # standardize the median column name (depends on the survey package
version)
1662  med_col <- setdiff(names(tab_med), c("Sexo","idade"))
1663  names(tab_med)[names(tab_med) == med_col] <- "mediana"
1664
1665  # 2) Unweighted n per cell (stable, via dplyr)
1666  counts_df <- Eligible_dataset %>%
1667    as.data.frame() %>%
1668    group_by(Sexo, idade) %>%
1669    summarise(n_unw = sum(!is.na(GRIP_STRENGTH)), .groups = "drop")
1670
1671  # 3) Merge and prepare for plotting
1672  plot_df <- tab_med %>%
1673    left_join(counts_df, by = c("Sexo","idade")) %>%
1674    mutate(
1675      n_unw = ifelse(is.na(n_unw), 0L, n_unw),
1676      idade = as.integer(idade)
1677    ) %>%
1678    filter(n_unw >= 5)       # optional: avoids very sparse points
1679
1680  # 4) DO NOT RUN, because the plot is very similar to the publication in
the other journal
1681  g1 <- ggplot(plot_df, aes(x = idade, y = mediana, color = Sexo)) +
1682    geom_point(size = 1.8, alpha = 0.9) +
1683    geom_smooth(method = "lm", se = FALSE, linewidth = 0.9) +
1684    labs(
1685      x = "Chronological Age",
1686      y = "median handgrip strength (kg)",
1687      title = "Força de preensão vs. idade (mediana ponderada por idade)",
1688      subtitle = "Pontos = mediana ponderada por idade; linha = tendência
linear por sexo"
1689    ) +
1690    theme_minimal() +
1691    theme(legend.position = "top")
1692
1693  g1
1694
1695
1696  # The panel below is the version selected to be used in Article
1697
1698
1699  library(ggplot2)
1700
1701  g2 <- ggplot(plot_df, aes(x = idade, y = mediana)) +
1702    geom_point(size = 1.8, alpha = 0.9) +
1703    geom_smooth(method = "lm", se = FALSE, linewidth = 0.9) +
1704    facet_wrap(
1705      ~ Sexo,
1706      ncol = 2,
1707      scales = "free_y",
1708      labeller = labeller(Sexo = c(masculino = "Men", feminino = "Women"))
1709    ) +
```

```
1710    scale_y_continuous(breaks = scales::breaks_width(5), minor_breaks =
NULL) +
1711    labs(x = "chronological age", y = "median handgrip strength (kg)") +
1712    theme_minimal(base_size = 12) +
1713    theme(
1714      text            = element_text(family = "Times New Roman", size = 10),
1715      axis.title.x    = element_text(family = "Times New Roman", size = 11),
1716      axis.title.y    = element_text(family = "Times New Roman", size = 11),
1717      axis.text.x     = element_text(family = "Times New Roman", size = 10),
1718      axis.text.y     = element_text(family = "Times New Roman", size = 10),
1719      strip.text      = element_text(family = "Times New Roman", size = 11,
face = "plain"),
1720      legend.title    = element_text(family = "Times New Roman", size = 12),
1721      legend.text     = element_text(family = "Times New Roman", size = 12),
1722      plot.title      = element_blank(),
1723      plot.subtitle   = element_text(family = "Times New Roman", size = 11),
1724      plot.caption    = element_text(family = "Times New Roman", size = 11)
1725    )
1726  g2
1727
1728  # The font was not switching to Times New Roman, so I asked ChatGPT for
help
1729  # I am testing below whether the exported output already shows the desired
font
1730
1731  library(svglite)
1732
1733  # export to SVG (vector), 12 x 8 cm
1734  svglite("FIGURE1.svg",
1735          width = 12/2.54, height = 8/2.54, bg = "white")
1736  print(g2)
1737  dev.off()
1738
1739  ######################################################################
1740  # Figure 1 (single panel): Men and Women on the same axes
1741  ######################################################################
1742
1743  # --- Figure 1 (single panel): shapes + inverted colors + legend inside
---
1744
1745  plot_df2 <- plot_df %>%
1746    mutate(
1747      Sexo_lab = factor(Sexo,
1748                        levels = c("masculino", "feminino"),
1749                        labels = c("Men", "Women"))
1750    )
1751
1752  # Common y-axis limits (rounded to multiples of 5)
1753  y_rng   <- range(plot_df2$mediana, na.rm = TRUE)
1754  y_lower <- floor(y_rng[1] / 5) * 5
1755  y_upper <- ceiling(y_rng[2] / 5) * 5
1756  if (y_upper == y_lower) y_upper <- y_lower + 5
1757
1758  g1_single <- ggplot(plot_df2, aes(x = idade, y = mediana, color =
Sexo_lab, shape = Sexo_lab)) +
1759    geom_point(size = 1.9, alpha = 0.9) +
1760    geom_smooth(method = "lm", se = FALSE, linewidth = 0.9) +
1761    scale_shape_manual(
1762      values = c("Men" = 17,  # triangle
1763                 "Women" = 16) # circle
1764    ) +
1765    # Inverted colors (edit as desired)
```

```
1766    scale_color_manual(
1767      values = c("Men" = "deepskyblue3",   # now Men = blue-ish
1768                 "Women" = "tomato3")      # now Women = red-ish
1769    ) +
1770    scale_y_continuous(
1771      breaks = seq(y_lower, y_upper, by = 5),
1772      minor_breaks = NULL
1773    ) +
1774    coord_cartesian(ylim = c(y_lower, y_upper)) +
1775    labs(
1776      x = "chronological age",
1777      y = "median handgrip strength (kg)",
1778      color = NULL,
1779      shape = NULL
1780    ) +
1781    theme_minimal(base_size = 12) +
1782    theme(
1783      text            = element_text(family = "Times New Roman", size = 10),
1784      axis.title.x    = element_text(family = "Times New Roman", size = 11),
1785      axis.title.y    = element_text(family = "Times New Roman", size = 11),
1786      axis.text.x     = element_text(family = "Times New Roman", size = 10),
1787      axis.text.y     = element_text(family = "Times New Roman", size = 10),
1788
1789      # Legend inside the plotting area (top-right)
1790      legend.position = c(0.97, 0.97),
1791      legend.justification = c(1, 1),
1792      legend.direction = "vertical",
1793      legend.background = element_rect(fill = "white", color = "grey70",
linewidth = 0.2),
1794      legend.key = element_rect(fill = "white", color = NA),
1795      legend.text = element_text(family = "Times New Roman", size = 10),
1796
1797      plot.title = element_blank()
1798    )
1799
1800  g1_single
1801
1802
1803  ########################################## This solution below was not used.
1804  ##### Solution 3 for visualizing the data: boxplot
1805  ##########################################
1806
1807  library(dplyr)
1808  library(survey)
1809  library(ggplot2)
1810
1811  # 1) Define age bands (e.g., 5-year groups). Adjust if you prefer.
1812  Medida_ok_df <- Eligible_dataset %>% as.data.frame() %>%
1813    mutate(idade_grp = cut(idade,
1814                           breaks = c(50,55,60,65,70,75,80,85,90, Inf),
1815                           labels = c("50–54","55–59","60–64","65–69","70–
74","75–79","80–84","85–89","90+"),
1816                           right = FALSE))
1817  # (optional) to match your methodological justification, you can focus on
<= 90 years:
1818  Medida_ok_df <- Medida_ok_df %>% filter(idade <= 90)
1819
1820  # 2) Back to the design
1821  des_grp <- Medida_ok_df %>% as_survey_design()
1822
1823  # 3) Weighted quantiles by Sexo x age band
1824  get_q <- function(p){
```

```
1825    out <- svyby(~GRIP_STRENGTH, ~Sexo + idade_grp, design = des_grp,
1826                 FUN = svyquantile, quantiles = p, ci = FALSE,
1827                 keep.var = FALSE, keep.names = FALSE, na.rm = TRUE)
1828    out <- as.data.frame(out)
1829    med_col <- setdiff(names(out), c("Sexo","idade_grp"))
1830    names(out)[names(out) == med_col] <- paste0("q", p*100)
1831    out
1832  }
1833  q25 <- get_q(0.25); q50 <- get_q(0.50); q75 <- get_q(0.75)
1834
1835  iqr_df <- q25 %>%
1836    left_join(q50, by = c("Sexo","idade_grp")) %>%
1837    left_join(q75, by = c("Sexo","idade_grp")) %>%
1838    arrange(Sexo, idade_grp)
1839
1840  # 4) Plot: “weighted boxplot” (IQR as a bar + median as a point)
1841  g_iqr <- ggplot(iqr_df, aes(x = idade_grp, group = 1)) +
1842    geom_linerange(aes(ymin = q25, ymax = q75), linewidth = 3, alpha = 0.35)
+
1843    geom_point(aes(y = q50), size = 2) +
1844    facet_wrap(~ Sexo, ncol = 2, scales = "free_y") +
1845    labs(x = "Faixa etária (anos)", y = "Força de preensão (kg)",
1846         title = "Distribuição ponderada da força por faixas etárias e
sexo",
1847         subtitle = "Barra = IQR (Q1–Q3), ponto = mediana ponderada") +
1848    theme_minimal(base_size = 12) +
1849    theme(axis.text.x = element_text(angle = 45, hjust = 1))
1850  g_iqr
1851
1852
1853  library(survey)
1854  library(dplyr)
1855
1856
1857
##############################################################################
1858  # ------------------------------------------------------------
1859  # Sex-specific designs with age ranges (to match your earlier runs)
1860  # Men: 50–85 | Women: 50–80
1861  # ------------------------------------------------------------
1862
##############################################################################
1863
1864  des_m <- subset(Eligible_dataset,
1865                  sexo == 1 &
1866                    idade >= 50 & idade <= 85 &
1867                    !is.na(idade) & !is.na(GRIP_STRENGTH))
1868
1869  des_f <- subset(Eligible_dataset,
1870                  sexo == 0 &
1871                    idade >= 50 & idade <= 80 &
1872                    !is.na(idade) & !is.na(GRIP_STRENGTH))
1873
1874  # ------------------------------------------------------------
1875  # Design-based linear models (survey-weighted)
1876  # ------------------------------------------------------------
1877
1878  fit_m <- svyglm(GRIP_STRENGTH ~ idade, design = des_m, family =
gaussian())
1879  fit_f <- svyglm(GRIP_STRENGTH ~ idade, design = des_f, family =
gaussian())
1880
```

```
cat("\n--- svyglm (Men, 50–85) ---\n")
print(summary(fit_m)$coefficients)
print(confint(fit_m))

cat("\n--- svyglm (Women, 50–80) ---\n")
print(summary(fit_f)$coefficients)
print(confint(fit_f))

# ------------------------------------------------------------
# Weighted R-squared (descriptive) using fitted values and weights
# Note: not a formal "design-based R^2", but a useful weighted GOF summary.
# ------------------------------------------------------------

weighted_r2 <- function(fit){
  y <- model.response(model.frame(fit))
  w <- weights(fit)
  yhat <- fitted(fit)

  ybar <- weighted.mean(y, w, na.rm = TRUE)
  sst  <- sum(w * (y - ybar)^2, na.rm = TRUE)
  sse  <- sum(w * (y - yhat)^2, na.rm = TRUE)

  1 - (sse / sst)
}

r2_m <- weighted_r2(fit_m)
r2_f <- weighted_r2(fit_f)

cat("\nWeighted R^2 (descriptive):\n")
cat("Men   (50–85):", round(r2_m, 3), "\n")
cat("Women (50–80):", round(r2_f, 3), "\n")

# MODEL FIT (descriptive):
# The survey-weighted linear model GRIP_STRENGTH ~ age shows a clear negative association,
# but age alone explains a limited share of individual variability. The weighted R^2 is 0.149
# for men (50–85) and 0.084 for women (50–80), indicating substantial dispersion around the
# mean age trend. Therefore, these linear models are used as a simple summary of the average
# decline with age rather than as high-accuracy predictive models.

# ------------------------------------------------------------
# Residual diagnostics (exploratory)
# ------------------------------------------------------------

diag_df_m <- data.frame(
  fitted = fitted(fit_m),
  resid  = residuals(fit_m, type = "response")
)

diag_df_f <- data.frame(
  fitted = fitted(fit_f),
  resid  = residuals(fit_f, type = "response")
)

plot(diag_df_m$fitted, diag_df_m$resid,
     xlab = "Fitted (Men)", ylab = "Residuals",
     main = "Residuals vs Fitted (Men, 50–85)")
abline(h = 0, lty = 2)
```

```
1938
1939  # DIAGNOSTIC NOTE (Men, 50–85):
1940  # The residuals-versus-fitted plot shows no strong curvature, suggesting
that a linear term in age
1941  # is a reasonable first-order approximation for the mean trend. However,
residual dispersion is
1942  # substantial, with a mild increase in variability at higher fitted values
and a few extreme residuals,
1943  # indicating limited predictive accuracy and possible
heteroscedasticity/heavy tails.
1944
1945
1946  plot(diag_df_f$fitted, diag_df_f$resid,
1947       xlab = "Fitted (Women)", ylab = "Residuals",
1948       main = "Residuals vs Fitted (Women, 50–80)")
1949  abline(h = 0, lty = 2)
1950
1951  # DIAGNOSTIC NOTE (Women, 50–80):
1952  # The residuals-versus-fitted plot shows no pronounced curvature,
supporting a linear age term as a
1953  # reasonable first-order approximation for the mean trend. Nonetheless,
residual variability is large,
1954  # with slightly wider spread at higher fitted values and a few extreme
residuals, suggesting possible
1955  # mild heteroscedasticity and heavy tails; thus, the model is best
interpreted as a summary of the
1956  # average decline rather than a high-accuracy predictive tool.
1957
1958
1959  qqnorm(diag_df_m$resid, main = "QQ-plot residuals (Men)");
qqline(diag_df_m$resid)
1960  # QQ-PLOT INTERPRETATION (Men, 50–85):
1961  # The normal Q–Q plot of the svyglm residuals suggests that normality is
reasonably
1962  # approximated in the center of the distribution (points close to the
reference line
1963  # around the median/near-zero quantiles). However, the points deviate from
the line at
1964  # both extremes: the left tail falls below the line and the right tail
rises above it.
1965  # This pattern indicates heavier-than-normal tails and/or the presence of
a few outlying
1966  # observations, i.e., more extreme negative and positive residuals than
expected under a
1967  # Gaussian error assumption. Therefore, the linear model can be retained
as a first-order
1968  # summary of the mean age trend, but residual behavior at the tails
reinforces that it has
1969  # limited individual-level predictive accuracy.
1970
1971
1972
1973  qqnorm(diag_df_f$resid, main = "QQ-plot residuals (Women)");
qqline(diag_df_f$resid)
1974  # QQ-PLOT INTERPRETATION (Women, 50–80):
1975  # Residuals follow the reference line closely in the center, but deviate
in the tails—most notably
1976  # in the upper tail (points above the line), indicating a heavier right
tail and a few extreme
1977  # positive residuals. Thus, normality is acceptable in the bulk but not in
the extremes.
1978
```

```
1979
1980
1981  ##########################################
1982  #### Subsetting by Brazilian region #####
1983  ##########################################
1984
1985  ####################
1986  # men by region
1987  ####################
1988
1989  #Northeast
1990  homens_Nordeste <- Eligible_dataset %>% select
(Idade2,sexo,GRIP_STRENGTH,Regiao) %>%
1991    filter(sexo == 1, Regiao == "Nordeste") %>%
1992    group_by(Idade2) %>%
1993    summarise(
1994      Nordeste = survey_mean(GRIP_STRENGTH,na.rm =TRUE, vartype = NULL)) %>%
1995    drop_na()
1996
1997  homens_Nordeste <- as_data_frame(homens_Nordeste)
1998
1999  #North
2000  homens_Norte <- Eligible_dataset %>% select
(Idade2,sexo,GRIP_STRENGTH,Regiao) %>%
2001    filter(sexo == 1, Regiao == "Norte") %>%
2002    group_by(Idade2) %>%
2003    summarise(
2004      Norte = survey_median(GRIP_STRENGTH,na.rm =TRUE, vartype = NULL)) %>%
2005    drop_na()
2006
2007  homens_Norte <- as_data_frame(homens_Norte)
2008
2009  #Central-West
2010  homens_Centro <- Eligible_dataset %>% select
(Idade2,sexo,GRIP_STRENGTH,Regiao) %>%
2011    filter(sexo == 1, Regiao == "Centro-Oeste") %>%
2012    group_by(Idade2) %>%
2013    summarise(
2014      Centro_Oeste = survey_median(GRIP_STRENGTH,na.rm =TRUE, vartype =
NULL)) %>%
2015    drop_na()
2016
2017  homens_Centro <- as_data_frame(homens_Centro)
2018
2019  #Southeast
2020  homens_Sudeste <- Eligible_dataset %>% select
(Idade2,sexo,GRIP_STRENGTH,Regiao) %>%
2021    filter(sexo == 1, Regiao == "Sudeste") %>%
2022    group_by(Idade2) %>%
2023    summarise(
2024      Sudeste = survey_median(GRIP_STRENGTH,na.rm =TRUE, vartype = NULL)) %>
%
2025    drop_na()
2026
2027  homens_Sudeste <- as_data_frame(homens_Sudeste)
2028
2029  #South
2030  homens_Sul <- Eligible_dataset %>% select
(Idade2,sexo,GRIP_STRENGTH,Regiao) %>%
2031    filter(sexo == 1, Regiao == "Sul") %>%
2032    group_by(Idade2) %>%
2033    summarise(
```

```
2034      Sul = survey_median(GRIP_STRENGTH,na.rm =TRUE, vartype = NULL)) %>%
2035    drop_na()
2036
2037  homens_Sul <- as_data_frame(homens_Sul)
2038
2039  #Saving the dataset for regional analysis among men
2040
2041  list_of_datasets <- list("Nordeste" = homens_Nordeste,
2042                           "Norte" = homens_Norte,
2043                           "Centro-Oeste" = homens_Centro,
2044                           "Sudeste" = homens_Sudeste,
2045                           "Sul" = homens_Sul)
2046
2047  write.xlsx(list_of_datasets, file = "dados_analise_regiao_male.xlsx")
2048
2049
2050  ##############################
2051  # women by region
2052  ##############################
2053
2054  #Northeast
2055  mulheres_Nordeste <- Eligible_dataset %>% select
(Idade2,sexo,GRIP_STRENGTH,Regiao) %>%
2056    filter(sexo == 0, Regiao == "Nordeste") %>%
2057    group_by(Idade2) %>%
2058    summarise(
2059      Nordeste = survey_median(GRIP_STRENGTH,na.rm =TRUE, vartype = NULL))
%>%
2060    drop_na()
2061
2062  mulheres_Nordeste <- as_data_frame(mulheres_Nordeste)
2063
2064  #North
2065  mulheres_Norte <- Eligible_dataset %>% select
(Idade2,sexo,GRIP_STRENGTH,Regiao) %>%
2066    filter(sexo == 0, Regiao == "Norte") %>%
2067    group_by(Idade2) %>%
2068    summarise(
2069      Norte = survey_median(GRIP_STRENGTH,na.rm =TRUE, vartype = NULL)) %>%
2070    drop_na()
2071
2072  mulheres_Norte <- as_data_frame(mulheres_Norte)
2073
2074  #Midwest
2075  mulheres_Centro <- Eligible_dataset %>% select
(Idade2,sexo,GRIP_STRENGTH,Regiao) %>%
2076    filter(sexo == 0, Regiao == "Centro-Oeste") %>%
2077    group_by(Idade2) %>%
2078    summarise(
2079      Centro_Oeste = survey_median(GRIP_STRENGTH,na.rm =TRUE, vartype =
NULL)) %>%
2080    drop_na()
2081
2082  mulheres_Centro <- as_data_frame(mulheres_Centro)
2083
2084  #Southeast
2085  mulheres_Sudeste <- Eligible_dataset %>% select
(Idade2,sexo,GRIP_STRENGTH,Regiao) %>%
2086    filter(sexo == 0, Regiao == "Sudeste") %>%
2087    group_by(Idade2) %>%
2088    summarise(
2089      Sudeste = survey_median(GRIP_STRENGTH,na.rm =TRUE, vartype = NULL)) %>
```

```
%
    drop_na()

  mulheres_Sudeste <- as_data_frame(mulheres_Sudeste)

  #South
  mulheres_Sul <- Eligible_dataset %>% select
(Idade2,sexo,GRIP_STRENGTH,Regiao) %>%
    filter(sexo == 0, Regiao == "Sul") %>%
    group_by(Idade2) %>%
    summarise(
      Sul = survey_median(GRIP_STRENGTH,na.rm =TRUE, vartype = NULL)) %>%
    drop_na()

  mulheres_Sul <- as_data_frame(mulheres_Sul)

  #Saving the dataset for regional analysis among men

  list_of_datasets2 <- list("Nordeste" = mulheres_Nordeste,
                            "Norte" = mulheres_Norte,
                            "Centro-Oeste" = mulheres_Centro,
                            "Sudeste" = mulheres_Sudeste,
                            "Sul" = mulheres_Sul)

  write.xlsx(list_of_datasets2, file = "dados_analise_regiao_female.xlsx")



  ############################################################
  # CALCULATING RELATIVE AGE
  ############################################################
  # RELATIVE AGE — MALE SEX (design-based revision)
  # 1) Standard population: predicted strength at ages 50..85 via survey-
weighted linear model (svyglm)
  # 2) Index population: read "dados_analise_regiao_male.xlsx" (Idade2 x
Region)
  # 3) Relative Age by region at the central ages 55,60,65,70,75 via linear
interpolation

  suppressPackageStartupMessages({
    library(dplyr); library(readxl); library(tidyr); library(purrr);
library(stringr)
    library(openxlsx); library(tibble)
  })

  # We now rely on the survey-weighted model fitted earlier for men:
  # fit_m <- svyglm(GRIP_STRENGTH ~ idade, design = des_m, family =
gaussian())
  stopifnot(exists("fit_m"))

  # 1) STANDARD POPULATION (Brazil, men) — based on svyglm coefficients
  co <- coef(fit_m)
  a_m <- unname(co[1])  # intercept
  b_m <- unname(co[2])  # slope for idade

  idades_padrao <- 50:85
  padrao_masc <- tibble(
    idade = idades_padrao,
    forca_pred = a_m + b_m * idades_padrao
  )

  # Sort by predicted strength (increasing) to ensure monotonic x for
```

```
inversion via approx()
  padrao_ord <- padrao_masc %>% arrange(forca_pred)

  # 2) INDEX POPULATION (Regions)
  arq_idx <- "dados_analise_regiao_male.xlsx"
  stopifnot(file.exists(arq_idx))
  sheets <- readxl::excel_sheets(arq_idx)

  # Central age of each band (Idade2)
  idade_central_map <- c(
    "53-57" = 55,
    "58-62" = 60,
    "63-67" = 65,
    "68-72" = 70,
    "73-77" = 75
  )

  indices_long <- map_dfr(
    sheets,
    function(sh) {
      df <- readxl::read_excel(arq_idx, sheet = sh)
      df <- as_tibble(df)
      names(df) <- gsub("\\s+", "_", names(df))

      # Identify the first numeric column as the strength column (region-
specific summary)
      num_cols <- names(df)[sapply(df, is.numeric)]
      if (length(num_cols) == 0 && ncol(df) >= 2) {
        # Coerce the 2nd column to numeric if needed
        df[[2]] <- suppressWarnings(as.numeric(df[[2]]))
        num_cols <- names(df)[2]
      } else {
        num_cols <- num_cols[1]
      }

      tibble(
        regiao = sh,
        Idade2 = as.character(df[[1]]),
        forca_indice = as.numeric(df[[num_cols]])
      )
    }
  ) %>%
    mutate(Idade2 = str_trim(Idade2)) %>%
    filter(Idade2 %in% names(idade_central_map)) %>%
    mutate(idade_central = unname(idade_central_map[Idade2])) %>%
    arrange(regiao, idade_central)

  # 3) RELATIVE AGE via linear interpolation (inverse mapping: age = f^{-1}
(strength))
  idade_relativa_from_y <- function(y, padrao_df) {
    approx(
      x = padrao_df$forca_pred,
      y = padrao_df$idade,
      xout = y,
      method = "linear",
      rule = 2   # linear extrapolation outside the predicted strength range
(set rule=1 for NA)
    )$y
  }

  idades_relativas <- indices_long %>%
    mutate(
```

```
2203      idade_relativa = idade_relativa_from_y(forca_indice, padrao_ord),
2204      delta_anos = idade_relativa - idade_central
2205    )
2206
2207  # 4) Export results (standard + relative ages)
2208  wb_out <- createWorkbook()
2209
2210  addWorksheet(wb_out, "Padrao_BR_masc")
2211  writeData(wb_out, "Padrao_BR_masc", padrao_masc)
2212
2213  addWorksheet(wb_out, "Relativas_Indices")
2214  out_tbl <- idades_relativas %>%
2215    select(regiao, Idade2, idade_central, forca_indice, idade_relativa,
delta_anos) %>%
2216    arrange(regiao, idade_central)
2217
2218  writeData(wb_out, "Relativas_Indices", out_tbl)
2219
2220  saveWorkbook(wb_out, "IdadeRelativa_Masculino.xlsx", overwrite = TRUE)
2221  message("Excel saved: ", normalizePath("IdadeRelativa_Masculino.xlsx"))
2222
2223
2224
2225  ############################################################
2226  # RELATIVE AGE — FEMALE SEX (design-based revision)
2227  # 1) Standard population (Brazil): predicted strength at ages 50..80 using
survey-weighted model (svyglm)
2228  # 2) Index population: medians by Idade2 x Region (Excel file)
2229  # 3) Relative Age by region at the central ages 55, 60, 65, 70, 75 via
linear interpolation
2230  ############################################################
2231
2232  suppressPackageStartupMessages({
2233    library(dplyr); library(readxl); library(tidyr); library(purrr);
library(stringr)
2234    library(openxlsx); library(tibble)
2235  })
2236
2237  # We now rely on the survey-weighted model fitted earlier for women:
2238  # fit_f <- svyglm(GRIP_STRENGTH ~ idade, design = des_f, family =
gaussian())
2239  stopifnot(exists("fit_f"))
2240
2241  # 1) STANDARD POPULATION (Brazil, women) — based on svyglm coefficients
2242  co_f <- coef(fit_f)
2243  a_f  <- unname(co_f[1])  # intercept
2244  b_f  <- unname(co_f[2])  # slope for idade
2245
2246  if (abs(b_f) < 1e-6) warning("Slope of fit_f is very close to zero;
relative age may become unstable.")
2247
2248  idades_padrao <- 50:80
2249  padrao_fem <- tibble(
2250    idade = idades_padrao,
2251    forca_pred = a_f + b_f * idades_padrao
2252  )
2253
2254  # Sort by predicted strength (increasing) for approx() with a monotonic x
2255  padrao_fem_ord <- padrao_fem %>% arrange(forca_pred)
2256
2257  # 2) INDEX POPULATION (Regions)
2258  arq_idx_f <- "dados_analise_regiao_female.xlsx"
```

```
2259  stopifnot(file.exists(arq_idx_f))
2260  sheets_f <- readxl::excel_sheets(arq_idx_f)
2261
2262  # Central age of each band (Idade2)
2263  idade_central_map <- c(
2264    "53-57" = 55,
2265    "58-62" = 60,
2266    "63-67" = 65,
2267    "68-72" = 70,
2268    "73-77" = 75
2269  )
2270
2271  indices_f_long <- map_dfr(
2272    sheets_f,
2273    function(sh) {
2274      df <- readxl::read_excel(arq_idx_f, sheet = sh)
2275      df <- as_tibble(df)
2276      names(df) <- gsub("\\s+", "_", names(df))
2277
2278      # Locate the first numeric column as the strength column (region-
specific summary)
2279      num_cols <- names(df)[sapply(df, is.numeric)]
2280      if (length(num_cols) == 0 && ncol(df) >= 2) {
2281        # Coerce the 2nd column to numeric if needed
2282        df[[2]] <- suppressWarnings(as.numeric(df[[2]]))
2283        num_col_name <- names(df)[2]
2284      } else {
2285        num_col_name <- num_cols[1]
2286      }
2287
2288      tibble(
2289        regiao = sh,
2290        Idade2 = as.character(df[[1]]),
2291        forca_indice = as.numeric(df[[num_col_name]])
2292      )
2293    }
2294  ) %>%
2295    mutate(Idade2 = str_trim(Idade2)) %>%
2296    filter(Idade2 %in% names(idade_central_map)) %>%
2297    mutate(idade_central = unname(idade_central_map[Idade2])) %>%
2298    arrange(regiao, idade_central) %>%
2299    drop_na(forca_indice)
2300
2301  # 3) RELATIVE AGE via linear interpolation (inverse mapping: age = f^{-1}
(strength))
2302  idade_relativa_from_y <- function(y, padrao_df) {
2303    approx(
2304      x = padrao_df$forca_pred,
2305      y = padrao_df$idade,
2306      xout = y,
2307      method = "linear",
2308      rule = 2   # linear extrapolation outside the predicted strength range
(set rule=1 for NA)
2309    )$y
2310  }
2311
2312  idades_relativas_f <- indices_f_long %>%
2313    mutate(
2314      idade_relativa = idade_relativa_from_y(forca_indice, padrao_fem_ord),
2315      delta_anos = idade_relativa - idade_central
2316    )
2317
```

```r
# 4) Export results (standard + relative ages)
wb_out_f <- createWorkbook()

addWorksheet(wb_out_f, "Padrao_BR_fem")
writeData(wb_out_f, "Padrao_BR_fem", padrao_fem)

addWorksheet(wb_out_f, "Relativas_Indices")
out_tbl_f <- idades_relativas_f %>%
  select(regiao, Idade2, idade_central, forca_indice, idade_relativa,
delta_anos) %>%
  arrange(regiao, idade_central)

writeData(wb_out_f, "Relativas_Indices", out_tbl_f)

saveWorkbook(wb_out_f, "IdadeRelativa_Feminino.xlsx", overwrite = TRUE)
message("Excel saved: ", normalizePath("IdadeRelativa_Feminino.xlsx"))


##############################################
# TRYING TO GENERATE comparative plots
##############################################

# ========================================================
# RELATIVE AGE PLOTS — BRAZILIAN REGIONS (Men/Women)
# Requires: IdadeRelativa_Masculino.xlsx and IdadeRelativa_Feminino.xlsx
#         (or objects idades_relativas / idades_relativas_f in the
environment)
# Output:  plotA (relative age vs chronological) and plotB (Δ years)
# ========================================================
suppressPackageStartupMessages({
  library(readxl); library(dplyr); library(tidyr)
  library(ggplot2); library(stringr); library(forcats)
})

# Load results (from file or from the environment)
load_rel <- function(path, sheet = "Relativas_Indices") {
  if (file.exists(path)) {
    readxl::read_excel(path, sheet = sheet) |>
      mutate(sexo = if (grepl("Femin", path, ignore.case = TRUE))
"feminino" else "masculino")
  } else {
    stop(paste("File not found:", path))
  }
}

df_m <- if (exists("idades_relativas")) {
  idades_relativas |> mutate(sexo = "masculino")
} else {
  load_rel("IdadeRelativa_Masculino.xlsx")
}

df_f <- if (exists("idades_relativas_f")) {
  idades_relativas_f |> mutate(sexo = "feminino")
} else {
  load_rel("IdadeRelativa_Feminino.xlsx")
}

rel_all <- bind_rows(df_m, df_f) |>
  rename(
    regiao = regiao,
    Idade2 = Idade2,
    idade_central = idade_central,
```

```
2377      forca_indice = forca_indice,
2378      idade_relativa = idade_relativa,
2379      delta_anos = delta_anos
2380    ) |>
2381    mutate(
2382      sexo = factor(sexo, levels = c("masculino","feminino"),
2383                    labels = c("Men","Women")),
2384      # Order regions as you prefer (below: South, Southeast, Midwest,
Northeast, North)
2385      regiao = fct_relevel(as.factor(regiao), "Sul","Sudeste","Centro-
Oeste","Nordeste","Norte"),
2386      idade_central = as.numeric(idade_central)
2387    ) |>
2388    filter(idade_central %in% c(55,60,65,70,75))
2389
2390  # Plot A — Relative age vs chronological age (by region)
2391  plotA <- ggplot(rel_all, aes(x = idade_central, y = idade_relativa, color
= regiao, group = regiao)) +
2392    geom_abline(slope = 1, intercept = 0, linetype = 3, linewidth = 0.7,
alpha = 0.8) +
2393    geom_line(linewidth = 1) +
2394    geom_point(size = 2.6) +
2395    facet_wrap(~ sexo, nrow = 1) +
2396    scale_x_continuous(breaks = c(55,60,65,70,75)) +
2397    labs(
2398      title = "Relative age by region vs chronological age",
2399      x = "Chronological age (years)",
2400      y = "Relative age (years)",
2401      color = "Region"
2402    ) +
2403    theme_classic(base_size = 12) +
2404    theme(legend.position = "bottom")
2405
2406  # Plot B — Δ years (relative age − chronological age)
2407  plotB <- ggplot(rel_all, aes(x = idade_central, y = delta_anos, color =
regiao, group = regiao)) +
2408    geom_hline(yintercept = 0, linetype = 3, linewidth = 0.7, alpha = 0.8) +
2409    geom_line(linewidth = 1) +
2410    geom_point(size = 2.6) +
2411    facet_wrap(~ sexo, nrow = 1) +
2412    scale_x_continuous(breaks = c(55,60,65,70,75)) +
2413    labs(
2414      title = "Difference in years (relative − chronological) by region",
2415      x = "Chronological age (years)",
2416      y = "Δ years",
2417      color = "Region"
2418    ) +
2419    theme_classic(base_size = 12) +
2420    theme(legend.position = "bottom")
2421
2422  # Display in R
2423  print(plotA)
2424  print(plotB)
2425
2426  # Save as PNG (high resolution)
2427  ggsave("plotA_relative_age_lines.png", plotA, width = 9, height = 4.2, dpi
= 300)
2428  ggsave("plotB_delta_lines.png",       plotB, width = 9, height = 4.2, dpi
= 300)
2429
2430  # B&W version for printing
2431  plotB_bw <- plotB +
```

```
2432    scale_color_grey(start = 0.2, end = 0.7)
2433  # ggsave("plotB_delta_lines_bw.png", plotB_bw, width = 9, height = 4.2,
dpi = 300)
2434
2435  ##############################################################################
2436  # Table 2: Median (SD) for HGS (kgf) by Age Group, Sex, and Region
2437  # + Export: Excel (median & SD) and Word (Table 2)
2438  ##############################################################################
2439
2440  suppressPackageStartupMessages({
2441    library(dplyr); library(tidyr); library(stringr)
2442    library(survey); library(srvyr)
2443    library(openxlsx)
2444    library(officer); library(flextable)
2445  })
2446
2447  # ------------------------------------------------------------
2448  # 0) Map of age groups to central ages (matching your Table 2)
2449  # ------------------------------------------------------------
2450  age_center_map <- c(
2451    "50-52" = 51, "53-57" = 55, "58-62" = 60, "63-67" = 65,
2452    "68-72" = 70, "73-77" = 75, "78-82" = 80
2453  )
2454
2455  # ------------------------------------------------------------
2456  # 1) Compute median (p50) and SD by Sex × Idade2 × Region (design-
weighted)
2457  #    -> create hgs_stats FIRST (so downstream blocks won't fail)
2458  # ------------------------------------------------------------
2459  hgs_stats <- Eligible_dataset %>%
2460    filter(!is.na(Sexo), !is.na(Idade2), !is.na(Regiao), !
is.na(GRIP_STRENGTH)) %>%
2461    mutate(
2462      Group = as.character(Idade2),
2463      idade_central = unname(age_center_map[Group])
2464    ) %>%
2465    filter(!is.na(idade_central)) %>%
2466    group_by(Sexo, Regiao, Group, idade_central) %>%
2467    summarise(
2468      hgs_median = as.numeric(survey_quantile(GRIP_STRENGTH, quantiles =
0.5, na.rm = TRUE, vartype = NULL))[1],
2469      hgs_sd     = sqrt(as.numeric(survey_var(GRIP_STRENGTH, na.rm = TRUE))
[1]),
2470      .groups = "drop"
2471    ) %>%
2472    # If there is more than 1 row per combination, collapse by mean
2473    group_by(Sexo, Regiao, Group, idade_central) %>%
2474    summarise(
2475      hgs_median = mean(hgs_median, na.rm = TRUE),
2476      hgs_sd     = mean(hgs_sd,     na.rm = TRUE),
2477      .groups = "drop"
2478    ) %>%
2479    # Standardize naming for downstream code
2480    rename(regiao = Regiao)
2481
2482  # ------------------------------------------------------------
2483  # 2) CALCULATING the SD of the median handgrip strength
2484  #    -> now safe because hgs_stats exists and has (Sexo, regiao,
idade_central)
2485  # ------------------------------------------------------------
2486
2487  # Duplicate diagnostics
```

```
dup_chk <- hgs_stats %>%
  count(Sexo, regiao, idade_central) %>%
  filter(n > 1)
if (nrow(dup_chk) > 0) {
  message("There are duplicates by (Sexo, regiao, idade_central). I will collapse by mean.")
  print(dup_chk)
}

# Collapse by mean if there are multiple rows per combination
hgs_stats_collapsed <- hgs_stats %>%
  group_by(Sexo, regiao, idade_central) %>%
  summarise(
    hgs_median = mean(hgs_median, na.rm = TRUE),
    hgs_sd     = mean(hgs_sd,     na.rm = TRUE),
    .groups = "drop"
  ) %>%
  mutate(
    hgs_median = round(hgs_median, 2),
    hgs_sd     = round(hgs_sd, 2)
  )

# Helper to generate a wide table WITHOUT values_fn
make_wide <- function(df, value_col) {
  df %>%
    select(idade_central, regiao, !!rlang::sym(value_col)) %>%
    group_by(idade_central, regiao) %>%
    summarise(value = mean(.data[[value_col]], na.rm = TRUE), .groups = "drop") %>%
    tidyr::pivot_wider(names_from = regiao, values_from = value) %>%
    arrange(idade_central)
}

# Final tables (one for median and one for SD for each sex)
hgs_wide_median_m <- hgs_stats_collapsed %>%
  filter(Sexo == "masculino") %>% make_wide("hgs_median")

hgs_wide_sd_m <- hgs_stats_collapsed %>%
  filter(Sexo == "masculino") %>% make_wide("hgs_sd")

hgs_wide_median_f <- hgs_stats_collapsed %>%
  filter(Sexo == "feminino") %>% make_wide("hgs_median")

hgs_wide_sd_f <- hgs_stats_collapsed %>%
  filter(Sexo == "feminino") %>% make_wide("hgs_sd")

# Export a clean Excel file
wb <- openxlsx::createWorkbook()
openxlsx::addWorksheet(wb, "HGS_median_Men");    openxlsx::writeData(wb, "HGS_median_Men",    hgs_wide_median_m)
openxlsx::addWorksheet(wb, "HGS_SD_Men");        openxlsx::writeData(wb, "HGS_SD_Men",        hgs_wide_sd_m)
openxlsx::addWorksheet(wb, "HGS_median_Women");  openxlsx::writeData(wb, "HGS_median_Women",  hgs_wide_median_f)
openxlsx::addWorksheet(wb, "HGS_SD_Women");      openxlsx::writeData(wb, "HGS_SD_Women",      hgs_wide_sd_f)
openxlsx::saveWorkbook(wb, "HGS_median_SD_by_group.xlsx", overwrite = TRUE)
message("Excel saved: ", normalizePath("HGS_median_SD_by_group.xlsx"))

# ------------------------------------------------------------
# 3) Build Table 2 with "median (SD)" — Word output
```

```
# ------------------------------------------------------------

# Translate region names to English and fix order to match your Table 2
region_map <- c(
  "Nordeste" = "Northeast",
  "Norte"    = "North",
  "Centro-Oeste" = "Midwest",
  "Sudeste"  = "Southeast",
  "Sul"      = "South"
)

hgs_en <- hgs_stats %>%
  mutate(
    Region = recode(as.character(regiao), !!!region_map),
    Region = factor(Region, levels =
c("Northeast","North","Midwest","Southeast","South"))
  )

# Helper to build "median (SD)" tables (wide) for one sex
make_display_table <- function(df_sex, digits_median = 1, digits_sd = 1) {
  df_sex %>%
    mutate(
      median_fmt = format(round(hgs_median, digits_median), nsmall =
digits_median, trim = TRUE),
      sd_fmt     = format(round(hgs_sd,     digits_sd),     nsmall =
digits_sd,     trim = TRUE),
      cell       = paste0(median_fmt, " (", sd_fmt, ")")
    ) %>%
    select(Group, idade_central, Region, cell) %>%
    arrange(idade_central, Region) %>%
    pivot_wider(names_from = Region, values_from = cell) %>%
    arrange(idade_central) %>%
    rename(`Central Age` = idade_central)
}

tab_men    <- hgs_en %>% filter(Sexo == "masculino") %>%
make_display_table()
tab_women  <- hgs_en %>% filter(Sexo == "feminino")  %>%
make_display_table()

doc <- read_docx()
doc <- body_add_par(doc, "Table 2. Median Hand Grip Strength (kgf) by Age
Group, Sex, and Region — Brazil", style = "heading 1")
doc <- body_add_par(doc, "Values are median (SD), design-weighted; HGS in
kgf. Central Age is the midpoint of the age group. Source: ELSI-Brasil (2016).",
style = "Normal")

ft_m <- flextable(tab_men) |> set_caption("Men") |> align(align =
"center", part = "all") |> theme_booktabs() |> autofit()
ft_w <- flextable(tab_women) |> set_caption("Women") |> align(align =
"center", part = "all") |> theme_booktabs() |> autofit()

doc <- body_add_flextable(doc, ft_m)
doc <- body_add_par(doc, "", style = "Normal")
doc <- body_add_flextable(doc, ft_w)

print(doc, target = "Table2_HGS_median_SD.docx")
message("Word saved: ", normalizePath("Table2_HGS_median_SD.docx"))

# ######################################################################
# Supplementary Table S1 — Relative age (years) by age group, sex, region
# + Supplementary Table S1 with Δ in ()
```

```
2595  ########################################################################
2596
2597  suppressPackageStartupMessages({
2598    library(dplyr); library(readxl); library(tidyr); library(stringr)
2599    library(forcats); library(officer); library(flextable)
2600  })
2601
2602  # --- S1: Relative age (years) ---
2603
2604  load_rel_simple <- function(path, sheet = "Relativas_Indices", sex_label =
c("Men","Women")) {
2605    if (file.exists(path)) {
2606      readxl::read_excel(path, sheet = sheet) |>
2607        dplyr::mutate(sexo = sex_label[1])
2608    } else if (exists("idades_relativas")) {
2609      idades_relativas |> dplyr::mutate(sexo = sex_label[1])
2610    } else {
2611      stop(paste("File not found and object does not exist:", path))
2612    }
2613  }
2614
2615  df_m <- load_rel_simple("IdadeRelativa_Masculino.xlsx", sex_label =
c("Men"))
2616  df_f <- load_rel_simple("IdadeRelativa_Feminino.xlsx",  sex_label =
c("Women"))
2617
2618  rel_all <- dplyr::bind_rows(df_m, df_f) |>
2619    dplyr::mutate(
2620      region = dplyr::recode(as.character(regiao),
2621                             "Nordeste" = "Northeast",
2622                             "Norte" = "North",
2623                             "Centro-Oeste" = "Midwest",
2624                             "Sudeste" = "Southeast",
2625                             "Sul" = "South",
2626                             .default = as.character(regiao)),
2627      region = forcats::fct_relevel(factor(region),
"Northeast","North","Midwest","Southeast","South"),
2628      Group = as.character(Idade2),
2629      `Central Age` = as.numeric(idade_central),
2630      value = round(as.numeric(idade_relativa), 1),
2631      sexo = factor(sexo, levels = c("Men","Women"))
2632    ) |>
2633    dplyr::filter(!is.na(`Central Age`), !is.na(value))
2634
2635  make_table <- function(df_sex) {
2636    df_sex |>
2637      dplyr::select(Group, `Central Age`, region, value) |>
2638      dplyr::arrange(`Central Age`, region) |>
2639      tidyr::pivot_wider(names_from = region, values_from = value) |>
2640      dplyr::arrange(`Central Age`)
2641  }
2642
2643  tab_men   <- rel_all |> dplyr::filter(sexo == "Men")   |> make_table()
2644  tab_women <- rel_all |> dplyr::filter(sexo == "Women") |> make_table()
2645
2646  doc <- read_docx()
2647  doc <- body_add_par(doc, "Supplementary Table S1. Relative age (years) by
age group (central age), sex, and region — Brazil", style = "heading 1")
2648  doc <- body_add_par(doc, "Values are relative ages (years). Central age is
the midpoint of each age group. Regions follow IBGE macro-regions.", style =
"Normal")
2649
```

```
ft_m <- flextable(tab_men)   |> set_caption("Men")   |> theme_booktabs() |
> align(align="center", part="all") |> autofit()
ft_w <- flextable(tab_women) |> set_caption("Women") |> theme_booktabs() |
> align(align="center", part="all") |> autofit()

doc <- body_add_flextable(doc, ft_m)
doc <- body_add_par(doc, "", style = "Normal")
doc <- body_add_flextable(doc, ft_w)

print(doc, target = "S1_Table_Relative_Age.docx")
message("Word saved: ", normalizePath("S1_Table_Relative_Age.docx"))

# --- S1: Relative age with Δ in () ---

SHEET_NAME <- "Relativas_Indices"
DECIMALS   <- 1

choose_col <- function(nms, opts, label){
  hit <- intersect(opts, nms)
  if (length(hit) == 0) stop(sprintf("Column '%s' not found. Options: %s",
label, paste(opts, collapse=", ")))
  hit[1]
}

load_rel <- function(path, sex_label) {
  if (!file.exists(path)) stop(paste("File not found:", path))
  df <- readxl::read_excel(path, sheet = SHEET_NAME)

  nms <- tolower(gsub("\\s+", "_", names(df)))
  names(df) <- nms

  col_reg   <- choose_col(nms, c("regiao","region"), "regiao/region")
  col_grp   <- choose_col(nms,
c("idade2","age_group","grupo","faixa","group"), "Idade2/age group")
  col_cage  <- choose_col(nms, c("idade_central","central_age"),
"idade_central/central_age")
  col_rage  <- choose_col(nms,
c("idade_relativa","relative_age","rel_age"), "idade_relativa/relative_age")

  df %>%
    dplyr::transmute(
      sexo          = sex_label,
      regiao        = .data[[col_reg]],
      Idade2        = as.character(.data[[col_grp]]),
      idade_central = as.numeric(.data[[col_cage]]),
      idade_relativa= as.numeric(.data[[col_rage]])
    )
}

df_m2 <- if (exists("idades_relativas")) {
  idades_relativas %>% dplyr::mutate(sexo = "Men")
} else {
  load_rel("IdadeRelativa_Masculino.xlsx", sex_label = "Men")
}

df_f2 <- if (exists("idades_relativas_f")) {
  idades_relativas_f %>% dplyr::mutate(sexo = "Women")
} else {
  load_rel("IdadeRelativa_Feminino.xlsx", sex_label = "Women")
}

rel_all2 <- dplyr::bind_rows(df_m2, df_f2) %>%
```

```
    dplyr::mutate(
      region = dplyr::recode(as.character(regiao),
                             "Nordeste"="Northeast","Norte"="North","Centro-
Oeste"="Midwest",
                             "Sudeste"="Southeast","Sul"="South",
                             .default = as.character(regiao)),
      region = forcats::fct_relevel(factor(region),
"Northeast","North","Midwest","Southeast","South"),
      Group         = as.character(Idade2),
      `Central Age` = as.numeric(idade_central),
      rel_age       = as.numeric(idade_relativa),
      delta         = rel_age - `Central Age`,
      val_fmt = format(round(rel_age, DECIMALS), nsmall = DECIMALS, trim =
TRUE),
      del_fmt = sprintf("%+.*f", DECIMALS, round(delta, DECIMALS)),
      cell    = paste0(val_fmt, " (", del_fmt, ")")
    ) %>%
    dplyr::filter(!is.na(`Central Age`), !is.na(rel_age)) %>%
    dplyr::arrange(sexo, `Central Age`, region)

  make_table2 <- function(df_sex) {
    df_sex %>%
      dplyr::select(Group, `Central Age`, region, cell) %>%
      tidyr::pivot_wider(names_from = region, values_from = cell) %>%
      dplyr::arrange(`Central Age`)
  }

  tab_men2   <- rel_all2 %>% dplyr::filter(sexo == "Men")   %>%
make_table2()
  tab_women2 <- rel_all2 %>% dplyr::filter(sexo == "Women") %>%
make_table2()

  doc <- read_docx()
  doc <- body_add_par(doc,
                      "Supplementary Table S1. Relative age (years) by age
group (central age), sex, and region — Brazil",
                      style = "heading 1")
  doc <- body_add_par(doc,
                      "Cells show relative age (years), with Δ (relative −
chronological) in parentheses. Positive Δ indicates older-appearing; negative Δ,
younger-appearing. Regions follow IBGE macro-regions.",
                      style = "Normal")

  ft_m2 <- flextable(tab_men2)   |> set_caption("Men")   |> theme_booktabs()
|> align(align="center", part="all") |> autofit()
  ft_w2 <- flextable(tab_women2) |> set_caption("Women") |> theme_booktabs()
|> align(align="center", part="all") |> autofit()

  doc <- body_add_flextable(doc, ft_m2)
  doc <- body_add_par(doc, "", style = "Normal")
  doc <- body_add_flextable(doc, ft_w2)

  print(doc, target = "S1_Table_Relative_Age_with_Delta.docx")
  message("Word saved: ",
normalizePath("S1_Table_Relative_Age_with_Delta.docx"))



  ##############################################
  #### Exploring GRIP_STRENGTH vs. age by region - 2 panels by sex
  #### GRIP_STRENGTH vs. age — 6-panel plot (5 regions + Brazil)
  #### (weighted linear regression using peso_calibrado)
```

```
#### - Ages limited to <= 85
### Page 1: Male (6 facets: Brazil + 5 regions) in 2 cols x 3 rows
#### Page 2: Female (same layout)
### - Adds a linear regression line (fit on the median-by-age points) + equation
##############################################


library(dplyr)
library(ggplot2)

# --- 0) Convert to data.frame (we will use weights directly) ---
df <- Eligible_dataset %>% as.data.frame()

# --- 1) Weighted median helper (no extra packages) ---
wtd_median <- function(x, w) {
  ok <- is.finite(x) & is.finite(w) & w > 0
  x <- x[ok]; w <- w[ok]
  if (length(x) == 0) return(NA_real_)
  o <- order(x)
  x <- x[o]; w <- w[o]
  cw <- cumsum(w) / sum(w)
  x[which(cw >= 0.5)[1]]
}

# --- 2) Region labels (Portuguese -> English) ---
region_map <- c(
  "Norte"        = "North",
  "Nordeste"     = "Northeast",
  "Sudeste"      = "Southeast",
  "Sul"          = "South",
  "Centro-Oeste" = "Midwest"
)

panel_levels <- c("Brazil", "North", "Northeast", "Southeast", "South", "Midwest")

# --- 3) Function: build one 6-facet plot for a given sex ---
make_plot_by_sex <- function(sex_value, sex_label) {

  # Base filter
  df_base <- df %>%
    filter(
      !is.na(idade),
      idade <= 85,
      !is.na(GRIP_STRENGTH),
      !is.na(peso_calibrado),
      !is.na(Regiao),
      !is.na(sexo),
      sexo == sex_value
    ) %>%
    mutate(
      Regiao_en = recode(as.character(Regiao), !!!region_map)
    )

  # (A) Region-specific: weighted median by age
  med_regions <- df_base %>%
    filter(!is.na(Regiao_en)) %>%
    group_by(Regiao_en, idade) %>%
    summarise(
      n_unw   = dplyr::n(),
      w_sum   = sum(peso_calibrado, na.rm = TRUE),
```

```
2817        med_grip = wtd_median(GRIP_STRENGTH, peso_calibrado),
2818        .groups = "drop"
2819      ) %>%
2820      mutate(Panel = Regiao_en)
2821
2822    # (B) Brazil overall: weighted median by age
2823    med_brazil <- df_base %>%
2824      group_by(idade) %>%
2825      summarise(
2826        n_unw   = dplyr::n(),
2827        w_sum   = sum(peso_calibrado, na.rm = TRUE),
2828        med_grip = wtd_median(GRIP_STRENGTH, peso_calibrado),
2829        .groups = "drop"
2830      ) %>%
2831      mutate(Panel = "Brazil")
2832
2833    # Combine and enforce panel order
2834    df_all <- bind_rows(med_brazil, med_regions) %>%
2835      mutate(Panel = factor(Panel, levels = panel_levels)) %>%
2836      filter(!is.na(Panel)) %>%
2837      arrange(Panel, idade)
2838
2839    # Regression equation per panel (fit on median-by-age points; weighted
by w_sum)
2840    eq_df <- df_all %>%
2841      group_by(Panel) %>%
2842      group_modify(~{
2843        fit <- lm(med_grip ~ idade, data = .x, weights = w_sum)
2844        b0 <- unname(coef(fit)[1])
2845        b1 <- unname(coef(fit)[2])
2846
2847        x_pos <- min(.x$idade, na.rm = TRUE) + 1
2848        y_pos <- max(.x$med_grip, na.rm = TRUE)
2849
2850        tibble(
2851          eq = paste0("y = ", round(b0, 2), " + ", round(b1, 3), "x"),
2852          x_pos = x_pos,
2853          y_pos = y_pos
2854        )
2855      }) %>%
2856      ungroup()
2857
2858    # Plot: 6 facets, 2 columns x 3 rows
2859    ggplot(df_all, aes(x = idade, y = med_grip)) +
2860      geom_point(alpha = 0.85, size = 1.2) +
2861      geom_smooth(method = "lm", se = FALSE, aes(weight = w_sum)) +
2862      geom_text(
2863        data = eq_df,
2864        aes(x = x_pos, y = y_pos, label = eq),
2865        inherit.aes = FALSE,
2866        hjust = 0, vjust = 1,
2867        size = 3.1
2868      ) +
2869      facet_wrap(~ Panel, ncol = 2, nrow = 3) +
2870      labs(
2871        x = "Age (years)",
2872        y = "Median GRIP_STRENGTH (kgf)",
2873        title = paste0("Median GRIP_STRENGTH vs Age (weighted median) — ",
sex_label, " (age ≤ 85)")
2874      ) +
2875      theme_bw()
2876  }
```

```
2877
2878  # --- Page 1: Male ---
2879  p_male <- make_plot_by_sex(sex_value = 1, sex_label = "Male")
2880  print(p_male)
2881
2882  # --- Page 2: Female ---
2883  p_female <- make_plot_by_sex(sex_value = 0, sex_label = "Female")
2884  print(p_female)
```
